# Molecular Structure and Conformation of Biodegradable Water-Soluble Polymers Control Adsorption and Transport in Model Soil Mineral Systems

**DOI:** 10.1021/acs.est.3c05770

**Published:** 2024-01-02

**Authors:** Kevin Kleemann, Patrick Bolduan, Glauco Battagliarin, Iso Christl, Kristopher McNeill, Michael Sander

**Affiliations:** † Institute of Biogeochemistry and Pollutant Dynamics, ETH Zurich, 8092 Zurich, Switzerland; ∥ BASF SE, Materials and Formulation Research, Carl-Bosch-Strasse 38, 67056 Ludwigshafen, Germany

**Keywords:** adsorption, biodegradable water-soluble polymers, plant protection formulations, soil minerals, transport

## Abstract

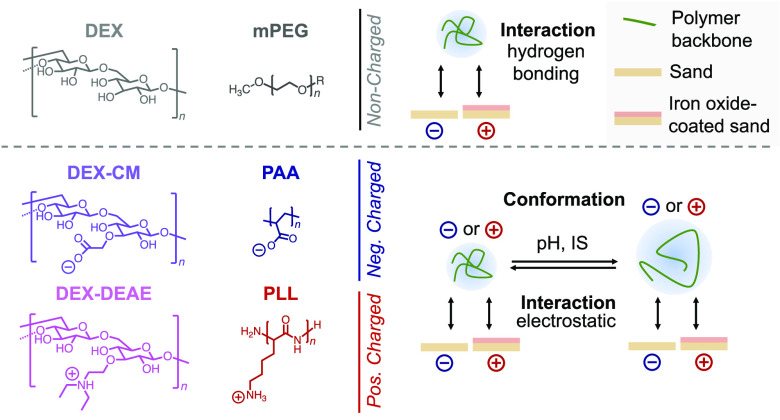

Water-soluble polymers (WSPs) are used in diverse applications, including agricultural formulations, that can result in the release of WSPs to soils. WSP biodegradability in soils is desirable to prevent long-term accumulation and potential associated adverse effects. In this work, we assessed adsorption of five candidate biodegradable WSPs with varying chemistry, charge, and polarity characteristics (i.e., dextran, diethylaminoethyl dextran, carboxymethyl dextran, polyethylene glycol monomethyl ether, and poly-l-lysine) and of one nonbiodegradable WSP (poly(acrylic acid)) to sand and iron oxide-coated sand particles that represent important soil minerals. Combined adsorption studies using solution-depletion measurements, direct surface adsorption techniques, and column transport experiments over varying solution pH and ionic strengths revealed electrostatics dominating interactions of charged WSPs with the sorbents as well as WSP conformations and packing densities in the adsorbed states. Hydrogen bonding controls adsorption of noncharged WSPs. Under transport in columns, WSP adsorption exhibited fast and slow kinetic adsorption regimes with time scales of minutes to hours. Slow adsorption kinetics in soil may lead to enhanced transport but also shorter lifetimes of biodegradable WSPs, assuming more rapid biodegradation when dissolved than adsorbed. This work establishes a basis for understanding the coupled adsorption and biodegradation dynamics of biodegradable WSPs in agricultural soils.

## Abstract 2

The fundamental understanding of adsorption of biodegradable water-soluble polymers to minerals advanced herein will enable fate and biodegradation assessment of these polymers in soils.

## Introduction

Water-soluble polymers (WSPs) have wide-ranging applications, many of which result in their release into the environment.^1−5^ A major application area is agricultural formulations,^6^ in which WSPs contribute to the application efficiency of crop protection chemicals and fertilizers. Using these formulations results in direct release of WSPs to agricultural soils, raising concerns of their accumulation to concentrations that may adversely impact soil fauna and flora, soil health, and ecosystem services. A promising strategy to overcome such accumulation is to replace stable, conventional WSPs with biodegradable alternatives (hereafter BWSPs) that undergo complete biodegradation in soils.^7,8^

Akin to the biodegradation of structural polymers and natural biopolymers,^9,10^ biodegradation of BWSP in soils is expected to be a two-step process: first, abiotic or extracellular enzymatic cleavage of the polymer backbone results in the formation of polymer-derived small molecules. Second, the small molecules are taken up by microorganisms and metabolized to produce energy under the formation of CO_2_ and microbial biomass. However, biodegradation of BWSPs likely is hampered by their adsorption to soil particle surfaces: such adsorptive stabilization has been demonstrated for natural biopolymers in soils, including nucleic acids and proteins,^11−13^ and was ascribed to microbial enzymes exhibiting decreased activity on surface-adsorbed substrates as compared to freely dissolved substrates. Concurrently, adsorption may retain the BWSPs in topsoils with higher microbial activity, which in turn, may enhance their biodegradation potential compared to that in deeper soil layers with lower microbial activity.^14,15^

Systematic adsorption studies of WSP and BWSP (which we jointly refer to as (B)WSP) in natural soils remain missing from the literature, reflecting that natural soils are highly heterogeneous and contain a variety of mineral and organic surfaces that challenge mechanistic interpretations of adsorption data. This challenge may be overcome by reductionistic experimental approaches in which adsorption is studied for selected, well-abundant soil minerals with defined surface chemistries. A reductionist approach was followed in two recent column transport studies on adsorption of diphenylmethyl polyethylene glycol to mixtures of quartz, illite, and goethite^13^ and of a PEG derivative and gum arabic to a sand and sandstone.^16,17^ The studies did, however, not systematically assess the adsorption of a larger set of (B)WSPs with diverse chemistries, including charged BWSPs.

Major candidate BWSPs for agricultural applications are (functionalized) polysaccharides, poly(ethylene glycol)s (PEGs), and polyamino acids. Some information on the adsorption of polysaccarides,^18−25^ PEGs^26−28^ and polyamino acids^29−35^ to model surfaces is available. These studies highlight that adsorption is dependent on the physicochemical properties of both the polymer (e.g., molecular weight, net charge, charge density, and ability to form hydrogen bonds) and the sorbent surface (e.g., charge, polarity, and hydrogen bond donor and acceptor sites). The literature suggests that adsorption of charged BWSPs to charged sorbent surfaces is mainly controlled by electrostatic interactions.^34,36^ With increasing adsorption and thus increasing BWSP sorbent surface coverage, repulsive electrostatic and steric interactions between adsorbed and dissolved BWSPs may slow down or even impair further adsorption despite unoccupied areas on the sorbent surface, a phenomenon referred to as the “excluded-area effect”.^37,38^

Electrostatic interactions are strongly modulated by the solution chemistry. Solution pH controls the charge density (and sign) of many BWSPs and sorbent surfaces. Increasing solution ionic strength (IS) not only leads to increased charge screening of polymer-sorbent electrostatic interactions but also attenuates intramolecular electrostatic repulsion between charged moieties within a BWSP molecule. As a result, charged BWSPs may adopt more compact conformations (i.e., a denser packing of folded strands of each polymer molecule) in adsorbed states,^39,40^ leading to higher maximum adsorbed concentrations at sorbent surface saturation.^35,41^ As compared to charged BWSPs, literature studies suggest that adsorption of uncharged BWSPs is mainly driven by H-bonding.^21,26,42^ Finally, in addition to the enthalpic contributions from electrostatic interactions and H-bonding, adsorption can be entropically favored since adsorption of the BWSP releases surface-coordinated molecules or ions from the sorbent surface into solution.^43,44^

This work provides a systematic assessment of adsorption of a larger set of candidate BWSPs (including charged and noncharged polymers) to sand and iron oxide-coated sand (IOCS) under varying solution conditions (i.e., pH and IS). We chose sand and IOCS because they are major soil mineral sorbents with pH-dependent variable surface charges (but net negative and positive surface charges at acidic to neutral pH, respectively).^45−47^ These two sorbents therefore allowed one to assess the effect of sorbent surface charge on (B)WSP adsorption. Based on the short experimental time frames and working with model sorbents in sterile-filtered solutions, we excluded BWSP biodegradation. The experiments were designed to test two hypotheses: First, electrostatics control adsorption of charged BWSP to sand and IOCS and increasing IS leads to more compact BWSP conformations and thereby higher maximum adsorbed concentrations at sorbent surface saturation. Second, H-bonding governs adsorption of uncharged BWSPs to sand and IOCS. We tested the first hypothesis by studying adsorption of positively charged poly-l-lysine (PLL) and diethylaminoethyl dextran (DEX-DEAE) and the negatively charged BWSP carboxymethyl dextran (DEX-CM) and the negatively charged nonbiodegradable WSP poly(acrylic acid) (PAA). We ran adsorption experiments at solution pH 5, 7, and 9 at a constant IS of 10 mM and, at pH 7, at IS of 10, 50, and 100 mM. We tested the second hypothesis by comparing adsorption of the uncharged dextran (DEX), which is both a hydrogen bond donor and acceptor, and polyethylene glycol monomethyl ether (mPEG), which only is hydrogen bond acceptor (i.e., the second −OH end group was capped by a fluorescent marker, see below, which we expect to have little if any effect on adsorption given the high molecular weight of the mPEG of 10 kDa). Adsorption to sand and IOCS was studied in solution-depleted batch reactors and in column transport experiments under saturated flow. To obtain molecular-level insights into the conformations of adsorbed BWSPs, we additionally used direct surface adsorption measurements to silica sensors using a quartz crystal microbalance with dissipation monitoring (QCM-D) and optical waveguide lightmode spectroscopy (OWLS). This work links (B)WSP chemistry and conformation sorbent characteristics of soil minerals, thereby providing a mechanistic basis for future studies elucidating the interplay between adsorption and biodegradation dynamics of BWSP in agricultural soils.

## Materials and Methods

### Chemicals

All chemicals were used as received (see Section S1 for details).

### Fluorescently Labeled (B)WSPs

We used fluorescently labeled (B)WSPs to allow for sensitive determination of (B)WSP concentrations using fluorescence measurements. Fluorescein isothiocyanate (FITC) diethylaminoethyl dextran (DEX-DEAE), FITC carboxymethyl dextran (DEX-CM), FITC dextran (DEX), and FITC poly-l-lysine (PLL) were purchased from Sigma-Aldrich (Merck KGaA, Germany). Poly(acrylic acid) (PAA) was purchased as sodium salt solution (45 wt % in H_2_O) from Sigma-Aldrich and covalently labeled with 6-amino fluorescein (AF) as described previously.^48^ FITC polyethylene glycol monomethyl ether (mPEG) was obtained from Creative PEGWorks. Key physicochemical properties of the (B)WSP are provided in Section S2.

### Sorbents

Silicon dioxide particles (hereafter “sand”) were purchased from Sigma-Aldrich (Switzerland; acid-washed and calcined, ≥99.7%). We sieved the sand in a vibratory sieve shaker (stainless steel sieves and AS200 basic, both from Retsch GmbH, Germany) to obtain 200 to 400 μm-sized particles. We subsequently cleaned this size fraction to remove impurities.^49^ The specific surface area (SSA) of the final sand used was 0.072 ± 0.002 m^2^g^–1^ determined by N_2_-BET analysis (ASAP 2020, Micromeritics; after 18–20 h of vacuum drying at 40 °C).

A subamount of the fractionated and cleaned sand was coated with iron oxide using the method of Mills et al.^50^ with slight modifications (details in Section S3). The N_2_-BET SSA (see above) of the IOCS was 0.398 ± 0.006 m^2^ g^–1^. The iron oxide coating made up 0.62 wt % of the IOCS, as determined using the phenanthroline assay^51^ (Section S3). We determined the pH-dependent surface charges and the point of zero charges of the sand and IOCS (Section S3).

### Solution Chemistry

All solutions were prepared using Milli-Q (MQ) water (resistivity >18 MΩ cm; Barnstead NANOpure Water Purification System). We tested IS at 10, 50, and 100 mM using NaCl and buffered the pH using *N*,*N*-diethyl piperazine (3 mM) for pH 5 and 9 and 3-(*N*-morpholino)propanesulfonic acid (3 mM) for pH 7. Both buffers are noncomplexing tertiary amines and are therefore expected to have no or at least minor interactions with the polymers and the mineral surfaces.^52^ We adjusted the pH by using 0.1 M NaOH or HCl.

### Solution-Depletion Batch Experiments

In 2 mL tubes (Eppendorf Protein-LoBind), we added 0.5 g of sand or IOCS and 1 mL of polymer solution to result in initial polymer concentrations of 20 μg mL^–1^ (sand) and 150 μg mL^–1^ (IOCS). The reaction tubes were shaken for 24 h in an overhead shaker (Heidolph REAX 2) at room temperature and 80 rpm. We subsequently centrifuged (Sigma 1-14 mini) the tubes at 6000 rpm for 10 min to sediment mineral particles. We then transferred 100 μL of supernatant solution into wells of black polypropylene 96-well plates, followed by fluorescence reading using a microplate reader (Tecan Infinite 200 PRO; excitation at 475 ± 9 nm; emission at 522 ± 20 nm; integration time of 20 μs).

The fluorescence intensity was converted to polymer concentrations by using fluorescence calibration curves. These curves were linear and covered the polymer concentration range needed for the given sorbent (i.e., up to 20 and 150 μg mL^–1^ for sand and IOCS experiments, respectively). For each (B)WSP, we ran sorbent-free controls with the same amount of (B)WSP added to determine and correct for polymer adsorption into the tube walls. Batch reactors were set up in duplicate. The adsorbed polymer concentration, Γ_Batch_ (μg cm^–2^), was calculated according to eq 1:
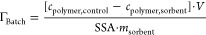
1where *c*_polymer, control_ (μg mL^–1^) is the final dissolved polymer concentration in sorbent-free control tubes, *c*_polymer, sorbent_ (μg mL^–1^) is the final dissolved polymer concentration in tubes containing either sand or IOCS, *V* (mL) is the solution volume, SSA (cm^2^ g^–1^) is the specific surface area of sand or IOCS, and *m*_sorbent_ (g) is the mass of sorbent in the tube.

### QCM-D and OWLS Measurements

We determined adsorption of PLL and DEX-DEAE to silica-coated sensors in QCM-D and OWLS. Detailed information on the measurement principle of both techniques and experimental setups is given in Sections S4 and S5. QCM-D measurements were conducted on an E4 system (Q-Sense AB, Sweden) with four individual flow-through cells operated in parallel, each holding a SiO_2_-coated sensor (QSX 303, Biolin Scientific). PLL and DEX-DEAE adsorption to the sensor decreased the resonance frequency *f* of the sensor fundamental tone (5 MHz) and overtones (*n*).^53^ The surface concentration, Γ_QCM-D_ (ng cm^–2^), of the polymer adlayer (i.e., the layer of adsorbed polymer molecules) was calculated from frequency shifts, Δ*f*_n_ (Hz), using the Sauerbrey equation (eq 2):^54,55^
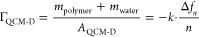
2where *k* (= 17.7 ng Hz^–1^ cm^–2^) is a sensor-specific proportionality constant, *m*_polymer_ (ng cm^–2^) is the polymer mass in the adlayer, *m*_water_ (ng cm^–2^) is the water mass coupling to the sensor’s oscillation, and *A*_QCM-D_ (cm^2^) is the sensor surface area. All data presented were calculated from the frequency shifts of the seventh overtone (i.e., *f*_7_ = 35 MHz). Γ_QCM-D_ provides an adlayer “wet” mass because the measurement principle includes mass contributions from adlayer water that couples to the oscillation of the sensor. The adsorbed mass of the adlayer was calculated by the Sauerbrey equation (eq 2). This equation was valid given that the adlayers were thin and showed little energy dissipation (i.e., the changes in energy dissipation, Δ*D*_7_, also determined by the instrument were small compared to the overtone normalized frequency shift (i.e., Δ*D*_7_/−Δ(*f*_7_/7) < 10^–7^ Hz^–1^).^55^

OWLS measurements were run on an OWLS 210 instrument (Microvacuum Ltd., Budapest, Hungary) equipped with a single laminar slit shear flow cell holding a planar optical waveguide coated with SiO_2_·TiO_2_ waveguiding layers of 160 nm thickness. For laterally uniform adlayers, the adsorbed concentration of the polymer, Γ_OWLS_ (ng cm^–2^), is given by eq 3:
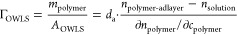
3where *m*_polymer_ (ng) is the adsorbed polymer mass in the adlayer, *A*_OWLS_ (cm^2^) is the sensor surface area, *d*_a_ (cm) is the adlayer thickness, *n*_polymer-adlayer_ (−) and *n*_solution_ (−) are the refractive indices of the polymer adlayer and of polymer-free solutions, respectively, calculated as previously described,^56^ and ∂*n*_polymer_/∂*c*_polymer_ (cm^3^ g^–1^) is the refractive index increment of the polymer (i.e., 0.16 cm^3^ g^–1^ for PLL^57^ and 0.15 cm^3^ g^–1^ for DEX-DEAE^58^). In contrast to QCM-D, OWLS is insensitive to adlayer water and thus provides the “dry” adlayer mass.

### Column Breakthrough Experiments

A detailed description of the setup is provided in Section S6. In brief, water-saturated columns packed with either sand or IOCS were freshly prepared for each experiment and mounted vertically. The inflow at the bottom of each column was connected to a syringe pump that delivered (B)WSP and nitrate solutions to the column with concentrations of *c*_inflow_(*t*). For each experiment, we measured *c*_inflow_(*t*) in the polymer solution before and after polymer delivery to the column by bypassing the column and thus delivering solution directly to the detector, assuming *c*_inflow_(*t*) remains constant during the breakthrough. The column outlet at the column top was connected to two optical flow-through cells for spectrophotometric absorbance and fluorescence intensity measurements to monitor the concentrations of the inert tracer nitrate and of the (B)WSPs in the column outflow (*c*_outflow_(*t*)), respectively. Each breakthrough experiment involved the following solution delivery steps: (i) (B)WSP-free solutions for >2 h to obtain stable baselines in outflow fluorescence intensity and UV absorption; (ii) (B)WSP-containing solutions for 3.5 h (= 34 column pore volumes (PVs)) (see Section S6 on determination); (iii) (B)WSP-free solutions for approximately ten PVs to rinse columns. In addition, we determined breakthrough of the inert tracer nitrate in each column, in one of duplicate columns prior to and in one after delivering (B)WSP. To this end, we delivered (iv) nitrate-containing solutions (0.1 mmol L^–1^), followed by (v) nitrate-free solutions, each for four PVs. Breakthrough curves (BTCs) of nitrate prior to and after delivering (B)WSPs were in very good agreement. For both (B)WSPs and nitrate, we calibrated the UV and fluorescence detectors using serially diluted solutions with known nitrate and (B)WSP concentrations, respectively. The responses on both detectors were linear with nitrate and (B)WSP concentrations over the tested concentration range.

Adsorbed (B)WSP concentrations on the sand and IOCS, Γ_column_ (ng cm^–2^), at the end of the adsorption step (see (ii) above) were calculated as the difference in the total (B)WSP mass delivered to the columns and the total mass in the column outflow according to eq 4:
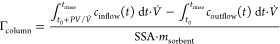
4where *V̇* (mL min^–1^) is the volumetric flow rate, PV (mL) is the pore volume of the column, *t*_0_ (min) and *t*_rinse_ (min) are the times at which we started to deliver (B)WSP solutions and initiated rinsing with (B)WSP-free solutions, respectively. For calculations, we corrected for the dead volumes in the tubing from the syringe pump to the column inlet and from the column outlet to the detectors.

### Numerical Modeling of Breakthrough Data

We fitted the BTCs of the (B)WSPs and of nitrate by the one-dimensional advection-dispersion equation (ADE) with an adsorption term under steady flow in water-saturated, packed columns according to eq 5:
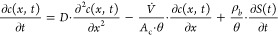
5where *c*(*x*, *t*) (μg mL^–1^) is the concentration of the (B)WSPs and nitrate, respectively, *t* (min) is time, *x* (cm) is distance along the column length, *D* (cm^2^ min^–1^) is the hydrodynamic dispersion coefficient, θ (−) is the porosity of the packed column, *A*_c_ (cm^2^) is the column cross-sectional area, ρ_b_ (g cm^–3^) is the bulk density of the sorbent (computed as ρ_b_ = (ρ_s_ (1 – θ))/θ; with material densities of sand and IOCS of ρ_s_ = 2.65 g cm^–3^), and  (μg g^–1^ min^–1^) is the adsorption rate of (B)WSP. We determined *D* by fitting BTCs of nitrate using eq 5 and setting the adsorption rate to zero (i.e., inert tracer). We modeled the BTCs of (B)WSPs with two adsorption rates in a two kinetic version of the Langmuir adsorption isotherm according to eq 6:
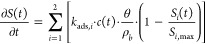
6where *k*_ads,*i*_ (min^–1^) is the adsorption rate constant for the two kinetic regimes *i* = 1 (fast) and 2 (slow) and *S*_*i*_ and *S*_*i*, max_ (μg g^–1^) are the actual adsorbed concentrations and maximum adsorbed (B)WSP concentrations at surface saturation for adsorption sites with kinetic *i*, respectively. The model fitted parameters were *k*_ads,1_, *k*_ads,2_, *S*_1, max_, and *S*_2, max_. All fits were performed using the Aquasim^59^ software (details in Section S7). The sorbent mass-normalized adsorbed concentrations *S*_*i*, max_, of the (B)WSPs were divided by SSA of the sorbent to obtain surface-normalized maximum adsorbed concentrations, Γ_*i*, max_ (ng cm^–2^).

### Theoretical Monolayer Coverage

We compared measured maximum adsorbed concentrations to theoretical maximum adsorbed concentrations calculated for two adsorption models (i.e., maximum hexagonal close packing (HCP) of (B)WSPs on the sorbent surface and random sequential adsorption (RSA) of (B)WSPs to the sorbent surface) according to eq 7:
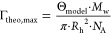
7where Γ_theo, max_ (ng cm^–2^) is the theoretical monolayer adsorbed surface concentration, *R*_h_ (here in units of (cm)) is the hydrodynamic radius of the (coiled) polymer, Θ_model_ (−) is the model-specific jamming limit (i.e., fraction of total surface area covered by polymer), *M*_w_ (here in units of ng mol^–1^) is the molecular weight of the polymer, and *N*_A_ (mol^–1^) is Avogadro’s number. For the charged polymers (i.e., DEX-DEAE, PLL, DEX-CM, and PAA), we used the assumption that *R*_h_ ≈ *M*_w_^0.5^, as previously demonstrated for strong polyelectrolytes at elevated salt concentrations.^60^ We found the assumption to be valid for DEX-DEAE based on comparing literature values of *R*_h_ and *M*_w_.^58,60^ For DEX and mPEG, we used literature values of *R*_h_ reported over a broad *M*_w_ range.^61,62^ The theoretical maximum surface coverages of (B) WSPs for the HCP model was  and for the RSA model Θ_RSA_ = 0.547.^38,63−65^ For calculations, we assumed that (B)WSPs adsorb as hard spheres onto flat and uniform surfaces. While the RSA scenario does not allow for mobility of adsorbed (B)WSP on the sorbent surface, the HCP scenario describes the densest possible (B)WSP packings that could result from their diffusion on the sorbent surface.^66^ More information about the models is provided in Section S8.

## Results and Discussion

### Theoretical Maximum Adsorbed (B)WSP Concentrations

For the charged (B)WSPs, the theoretical maximum adsorbed surface concentrations for Γ_HCP, max_ and Γ_RSA, max_ were 48 and 29 ng cm^–2^, respectively. We note that all charged polymers tested resulted in a single Γ_HCP, max_ and Γ_RSA, max_ value because we assumed *R*_h_ ≈ *M*_w_^0.5^ (see previous section), resulting in eq 7 being independent of *M*_w_. For the uncharged BWSPs, Γ_HCP, max_ and Γ_RSA, max_ values were 60 and 44 ng cm^–2^ for DEX, respectively, and 91 and 51 ng cm^–2^ for mPEG, respectively. The theoretical maximum adsorbed surface concentrations are calculated assuming adsorbed (B)WSPs on an “inert”, flat sorbent surface and thus do not consider effects of sorbent surface chemistry. In all batch adsorption experiments below, we added (B)WSPs in amounts that were in excess to these theoretical maximum adsorbed concentrations to ensure that adsorption could not be limited by the added (B)WSPs amounts.

### Adsorption of (B)WSPs to Sand

#### Acid–Base Titrations

The sand surface was negatively charged over the entire tested pH range, as determined by acid–base titrations (Section S3).

#### Batch Adsorption Experiments

**Figure 1:**
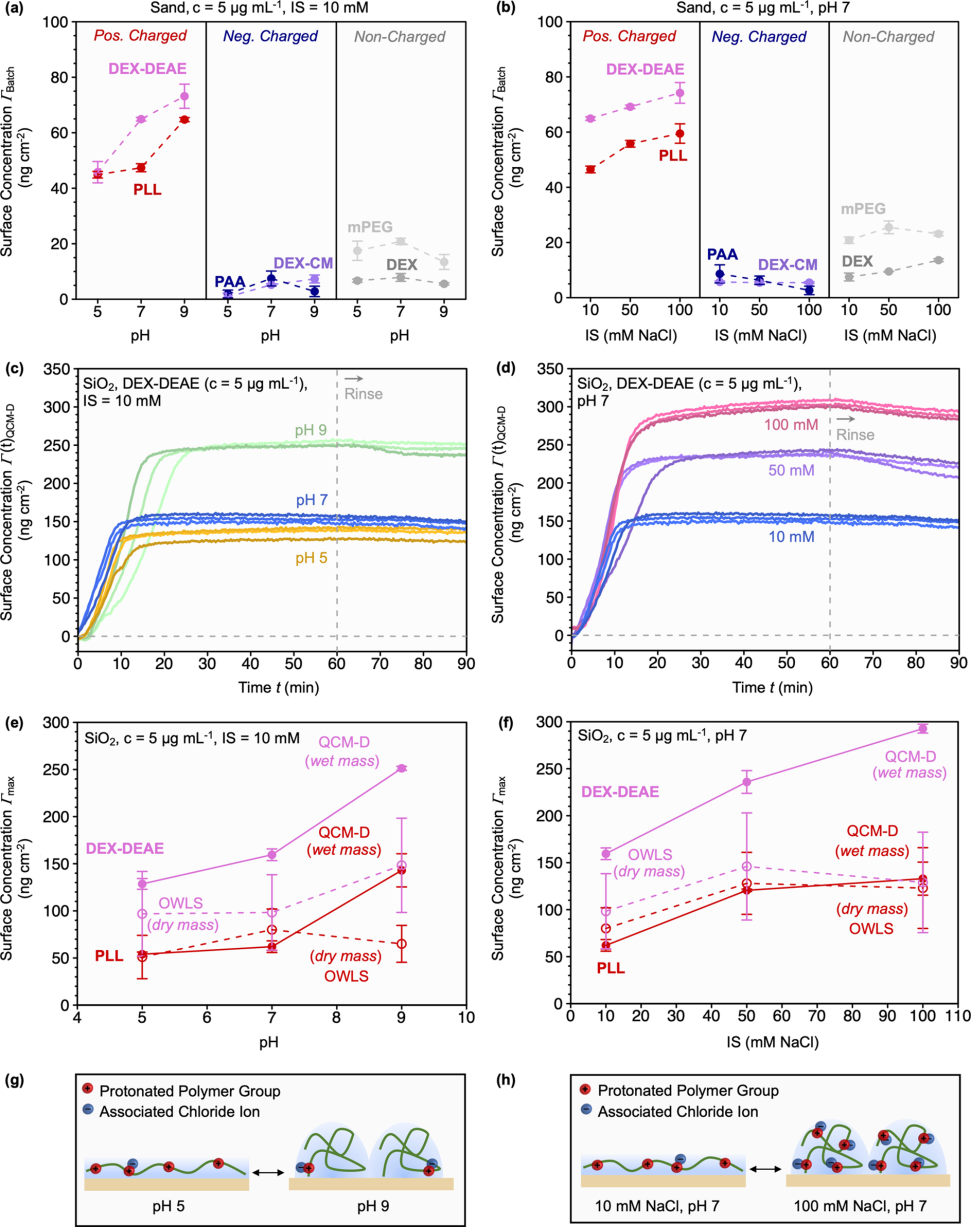
Adsorption of selected water-soluble polymers to sand and silica surfaces measured in batch equilibration studies (panels a, b), by quartz-crystal microbalance with dissipation monitoring (QCM-D) (panels c–f) and optical waveguide laser spectroscopy (OWLS) (panels g, h). The polymers are diethylaminoethyl dextran (DEX-DEAE), poly-l-lysine (PLL), carboxymethyl dextran (DEX-CM), poly(acrylic acid) (PAA), dextran (DEX), and polyethylene glycol monomethyl ether (mPEG). (a, b) Effects of solution pH (a) and ionic strength (b) (IS; all at pH 7) on adsorbed concentrations Γ_batch_ of water-soluble polymers on sand. The data points and error bars represent the mean values and ranges of duplicate batch reactors run for each tested condition. (c, d) Changes in adsorbed concentrations of DEX-DEAE on silica-coated QCM-D sensors over time as a function of pH (panel c) and IS (panel d). (e, f) Effects of solution pH (panel e) and IS (panel f) on the maximum adsorbed concentrations of DEX-DEAE and PLL on silica coated QCM-D and OWLS sensors. (g, h) Schematic depiction of suggested changes in adlayer architecture as a function of solution pH (panel g) and IS (panel h).

Over the tested pH range, adsorbed surface concentrations Γ_batch_ on sand were the highest for positively charged DEX-DEAE and PLL (Γ_batch_ = 45 to 75 ng cm^–2^), followed by noncharged mPEG and DEX (from 7 to 20 ng cm^–2^) and by negatively charged PAA and DEX-CM (<10 ng cm^–2^) (Figure 1a). Adsorbed concentrations of DEX-DEAE and PLL increased with increasing pH from 5 to 9 and were in the range of Γ_HCP, max_, suggesting complete surface coverage of the sand by the two BSWPs. By comparison, pH had little effect on the adsorption of the other (B)WSPs to sand. Increasing IS from 10 to 50 and 100 mM at pH 7 resulted in increasing DEX-DEAE and PLL adsorption to the sand (Figure 1b). Conversely, increasing the IS had only minor effects on the adsorption of the uncharged and negatively charged (B)WSPs.

The observed adsorption differences for the tested (B)WSP support the hypothesized importance of electrostatic interactions and H-bonding between the (B)WSPs and the sand surface. Pronounced PLL and DEX-DEAE adsorption is consistent with their electrostatic attraction to the sand surface, while minor adsorption of PAA and DEX-CM likely reflected their electrostatic repulsion from the sand surface. Intermediate adsorption extents of mPEG and DEX likely resulted from weak H-bond interactions due to few Brønsted acid sites on the sand over the tested pH range.^26^

PLL and DEX-DEAE are less positively charged at pH 9 due to partial deprotonation of their cationic ammonium groups (p*K*_a_ close to 10 in PLL^67^ and p*K*_a1_ = 5.5 and p*K*_a2_ = 9.2 for DEX-DEAE, as determined by acid–base titration; Section S2). Increasing IS increased shielding of the charged groups along the polymer chain and thus decreased intramolecular electrostatic repulsion. As a result, DEX-DEAE and PLL likely adopted more compact conformations in solution and in adsorbed states. Increasing IS likely also decreased electrostatic repulsion of neighboring (B)WSP molecules in adsorbed states, thereby allowing for denser packings (i.e., decreased excluded-area effects).^35,37,38,68,69^

#### QCM-D and OWLS Experiments

We conducted complementary QCM-D and OWLS experiments to support more compact conformations and denser packings of DEX-DEAE and PLL in adsorbed states at high pH and IS. Figure 1c,d shows representative QCM-D results for DEX-DEAE adsorption as functions of pH and IS, respectively. The corresponding OWLS data for DEX-DEAE adsorption as well as the QCM-D and OWLS data for PLL adsorption are provided in Sections S4 and S5, respectively.

Following sensor equilibration to BWSP-free solutions at *t* < 0 min, delivering DEX-DEAE solutions to the QCM-D sensors resulted in immediate and pronounced DEX-DEAE adsorption, as evidenced from increasing Γ(*t*)_QCM-D_ (Figure 1c,d). Adsorbed masses then sharply transitioned into plateaus, indicative of sensor surface saturation. Rinsing with DEX-DEAE-free solutions beyond *t* = 60 min resulted in very small decreases in the adsorbed masses, implying irreversible adsorption. Consistent with the results of batch experiments, adsorbed masses of DEX-DEAE increased from pH 5 and 7 to 9 and from 10 to 50 and 100 mM IS (Figure 1c,d).

Figure 1e,f shows final (i.e., plateaued) adsorbed “wet” (QCM-D) and “dry” (OWLS) DEX-DEAE and PLL concentrations on the silica sensor surfaces as a function of pH and IS, respectively. The “wet” mass determined by QCM-D is higher as it has mass contributions not only from the adsorbed polymer but also from water associated with and hydrodynamically coupling to the polymer adlayer during the sensor oscillation. The water contributions typically vary between 20 and 50 wt % of the total “wet” adlayer mass depending on the adlayer architecture and thickness.^36^ The PLL and DEX-DEAE concentrations adsorbed in OWLS experiments were slightly higher than those determined in batch equilibration experiments. More importantly, and consistent with the batch experiments, DEX-DEAE and PLL adsorption to silica sensors increased from pH 5 and 7 to 9 and with increasing IS at pH 7 (Figure 1e,f), with the exception being pH-independent PLL adsorption in OWLS. The increases in DEX-DEAE and PLL adsorption with pH and IS in the surface adsorption experiments thus strongly support that BWSPs adsorbed in more compact conformations and denser packings on silica surfaces at pH 9 and IS = 100 mM.

A comparison of QCM-D and OWLS data provides additional molecular-level insight into the architecture of PLL and DEX-DEAE adlayers. Good agreement of final “wet” and “dry” PLL adsorbed concentrations at pH 5 and 7 and at all tested ISs at pH 7 suggests that PLL adsorbed in flat and elongated conformations with only minor mass contributions of coupling water to the total adlayer mass. Conversely, at pH 9, higher “wet” than “dry” adsorbed PLL concentrations implied that the PLL adlayer contained hydrodynamically coupled water, consistent with more compact and coiled PLL conformations and higher PLL adlayer thicknesses (Figure 1g). As compared to PLL, DEX-DEAE adlayers exhibited hydrodynamically coupled water under all solution conditions, with the highest adlayer water contents at pH 9. The higher water contents of DEX-DEAE than PLL adlayers suggested more coiled conformations of DEX-DEAE molecules, presumably due to stronger intra- and intermolecular H-bonding in and between DEX-DEAE molecules. Higher water contents in the adlayer likely also resulted from the branched structure of DEX-DEAE compared to the linear PLL. We schematically depicted the suggested changes of DEX-DEAE and PLL adlayer architectures with increasing pH and IS in Figure 1g,h.

#### Column Breakthrough Experiments

We studied DEX-DEAE and PLL adsorption to sand under flow conditions. We constrained column experiments to these two BWSPs as all other (B)WSPs showed only weak adsorption to sand in batch equilibration experiments. As expected, breakthrough of the inert tracer nitrate through the packed column was complete (i.e., nitrate concentrations in the column effluent matched the inflow concentrations; *c*_outflow_/*c*_inflow_ = 1) within two PVs of delivering nitrate solutions to the columns (see Section S6), consistent with no nitrate adsorption to the sand.

**Figure 2:**
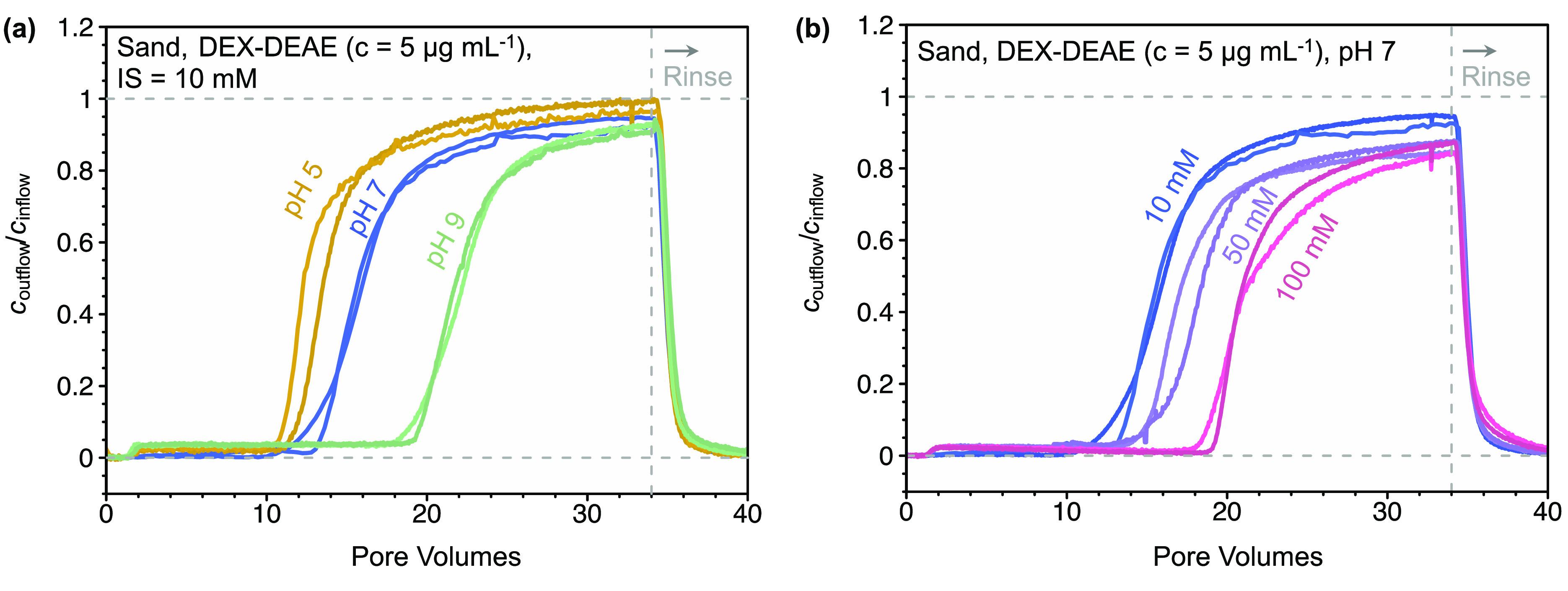
Results of column breakthrough experiments of diethylaminoethyl dextran (DEX-DEAE) in columns filled with sand. (a) Effect of solution pH on DEX-DEAE breakthrough, with *c*_outflow_ and *c*_inflow_ corresponding to the concentrations of DEX-DEAE in the column outflow and inflow, respectively. (b) Effect of solution ionic strength (IS in (mM NaCl)) on DEX-DEAE breakthrough, all at pH 7.

Upon delivering BWSP-containing solutions to the columns starting at *t* = 0 min, breakthrough of DEX-DEAE (Figure 2a,b) and PLL (Section S6) occurred between 10 and 20 PVs and thus was retarded under all tested solution conditions. This retardation implied substantial DEX-DEAE and PLL adsorption to the sand particles. At the end of the BWSP delivery step at 34 PVs, *c*_outflow_/*c*_inflow_ either reached or approached values of unity. Upon rinsing with BWSP-free solutions after 34 PV, *c*_outflow_/*c*_inflow_ values sharply decreased within only a few PVs of rinsing, implying that there was little PLL and DEX-DEAE desorption from the sand particles, and hence, adsorption was highly irreversible. This finding is consistent with the QCM-D and OWLS data, which showed little DEX-DEAE and PLL desorption upon sensor rinsing (e.g., Figure 1c,d). Irreversible adsorption is consistent with strong electrostatic attraction of DEX-DEAE and PLL to the sand and, hence, a low probability of simultaneous breaking all contact points between the BWSP and the sand surface to allow for surface detachment.^36^

Adsorbed concentrations of DEX-DEAE and PLL on the sand at the end of the adsorption step increased with both solution pH values from 5 to 7 and 9 and at pH 7 with increasing IS (Figure S9). The adsorbed concentrations ranged from 40 to 85 ng cm^–2^ for DEX-DEAE and from 35 to 70 ng cm^–2^ for PLL and were thus in good agreement with adsorbed concentrations of the same polymers determined in batch experiments and in the range of estimated Γ_HCP, max_. This finding suggests that results from batch experiments can be used to predict final adsorbed concentrations of the same polymers to the same sorbent (here: sand) under flow, which more closely mimics conditions during water movement in soils.

All BTCs of DEX-DEAE and PLL showed two distinct phases: in the first phase of breakthrough, *c*_outflow_/*c*_inflow_ increased sharply to values between 0.6 and 0.8. In the subsequent second phase, the BTCs flattened with *c*_outflow_/*c*_inflow_ values increasing more slowly toward a ratio of unity. At high pH and IS, this second phase was more pronounced (particularly for PLL at pH 9; see Figure S7) and resulted in incomplete breakthrough when we initiated rinsing after 34 PVs. Similar shapes of BTCs were previously reported for transport of gum arabic, surfactants, and fullerenes to sand or sediment and were ascribed to slow kinetic adsorption.^17,70−72^ Thus, the second phase for breakthrough of DEX-DEAE and PLL indicated that their adsorption to sand had a slow kinetic component, as discussed in more detail below.

### Adsorption of (B)WSPs to IOCS

#### Acid–Base Titrations

The IOCS carried a net positive charge from pH 4.6 to above pH 7, with surface charge densities decreasing with increasing pH (see the acid–base titration data in Section S3). At pH 7.6, the IOCS had a zero net charge, in good agreement with the reported point of zero charge (pH_PZC_) of goethite (i.e., pH_PZC_ = 6.6–8.6^73^) and with reported acidity constants for protonated iron surface hydroxy FeOH_2_^+1/2^ groups (i.e., p*K*_a_ = 7.7^74^). At pH > 7.6, the IOCS carried a net negative charge that increased with increasing pH. The surface charge characteristics of the IOCS were thus dominated by the iron oxide coating, given that the sand used for coating was net negatively charged over the entire tested pH range (see above). The approximately 5.5-fold higher specific surface area of the IOCS compared to sand suggests extensive iron oxide coating. Yet, we cannot exclude that a small fraction of the sand surface remained bare and not coated by the iron oxides.

#### Batch Equilibration Experiments

**Figure 3:**
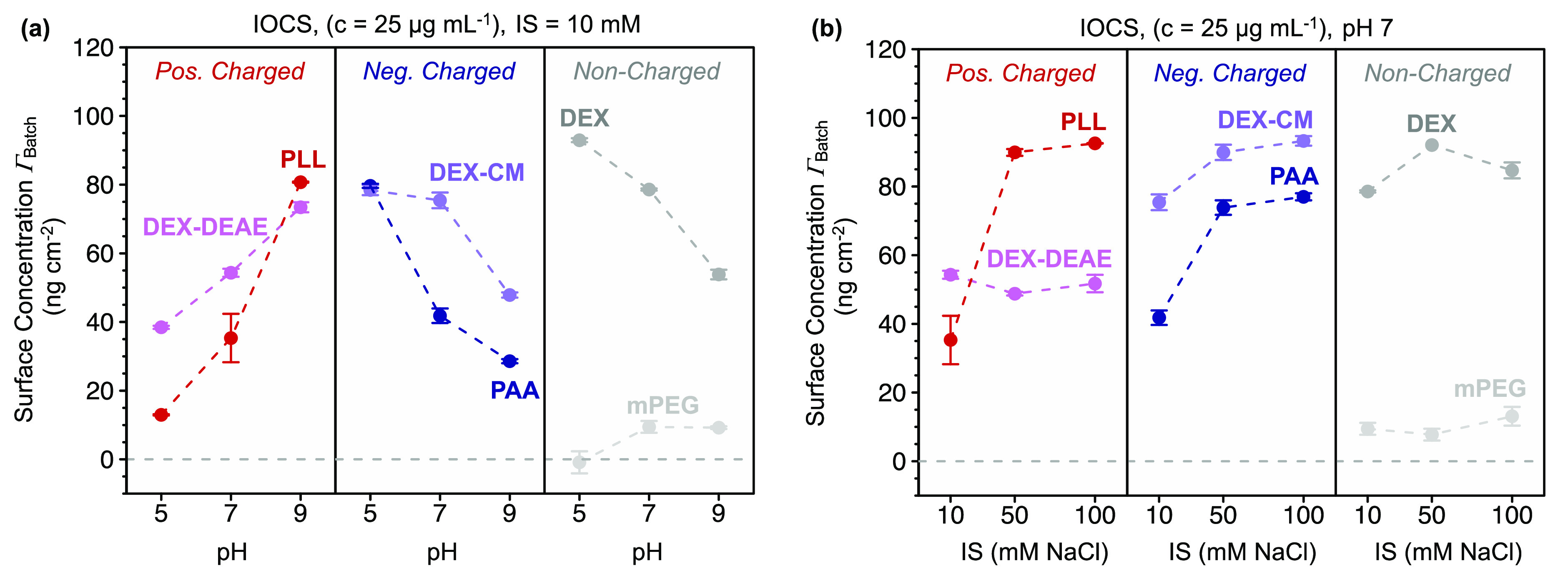
Adsorbed concentrations of water-soluble polymers to the surface of iron oxide-coated sand (IOCS) as determined by batch equilibration studies. The water-soluble polymers are diethylaminoethyl dextran (DEX-DEAE), poly-l-lysine (PLL), carboxymethyl dextran (DEX-CM), poly(acrylic acid) (PAA), dextran (DEX), and polyethylene glycol monomethyl ether (mPEG). Changes in the final adsorbed concentration as a function of (a) pH (at constant ionic strength, IS = 10 mM) or (b) IS (at constant pH 7). The data points and error bars represent the mean values and absolute ranges of duplicate measurements, respectively.

Adsorption to IOCS of all (B)WSPs except mPEG was extensive under the tested solution conditions, with adsorbed concentrations ranging from theoretical Γ_RSA, max_ to values higher than Γ_HCP, max_. Adsorption of DEX-DEAE and PLL to IOCS increased with increasing pH from approximately Γ_batch_ = 40 to 75 ng cm^–2^ and from 15 to 80 ng cm^–2^, respectively (Figure 3a). Negatively charged DEX-CM and PAA showed pronounced adsorption to IOCS, with Γ_batch_ decreasing with increasing pH from approximately 80 ng cm^–2^ for DEX-CM and PAA at pH 5 to 45 and 30 ng cm^–2^ for DEX-CM and PAA at pH 9, respectively. While there was only weak adsorption of H-bond accepting mPEG, H-bond donating and accepting DEX adsorbed strongly and showed decreasing Γ_batch_ with increasing pH. Increasing IS from 10 to 50 and 100 mM at pH 7 increased adsorption of PLL, DEX-CM, and PAA but not of DEX-DEAE (Figure 3b). Adsorption of uncharged DEX and mPEG was little affected by IS.

Increasing PLL and DEX-DEAE adsorption to IOCS with increasing pH from 5 to 7 can be rationalized by decreasing electrostatic repulsion of these two BWSPs from the IOCS and, at pH 9, by electrostatic attraction. Adsorption of PLL and DEX-DEAE to IOCS at pH 5 and 7 could have also occurred to patches of bare sand provided that not the entire sand surface was coated by iron oxides. It is conceivable that increasing PLL adsorption with increasing IS reflected increasing attenuation of the PLL electrostatic repulsion from IOCS. However, this IS effect was not observed for DEX-DEAE adsorption. The reason for different IS dependencies of the adsorption of PLL and DEX-DEAE to IOCS remains unclear.

Decreasing DEX-CM and PAA adsorption with increasing pH is consistent with decreasing electrostatic attraction from pH 5 to 7 and net electrostatic repulsion from the IOCS at pH 9. At pH 7, increasing adsorption of DEX-CM and PAA with increasing IS likely resulted from these (B)WSPs adapting more coiled and thus compact conformations and denser packings in adsorbed states due to attenuated intra- and intermolecular electrostatic repulsion at high IS (see the above discussion).

Low mPEG adsorption to IOCS under all pH and IS conditions suggests only weak H-bonding between H-bond donating sites on the IOCS surface and the H-bond accepting ether moieties in mPEG.^26^ By comparison, the strong adsorption of DEX under all solution conditions is likely caused by strong H-bonding between the H-bond donating and accepting sites of DEX and the IOCS.^75^ This finding suggests that H-bonding may also have contributed to the adsorption of DEX-CM and DEX-DEAE to the IOCS. The decrease in DEX adsorption with increasing pH suggests preferred hydrogen H-bonding with FeOH_2_^+1/2^ over FeOH^–1/2^ moieties (abundance approximately 0.1–0.5 sites nm^–2^, calculated from acid–base titrations).

#### Column Experiments

**Figure 4:**
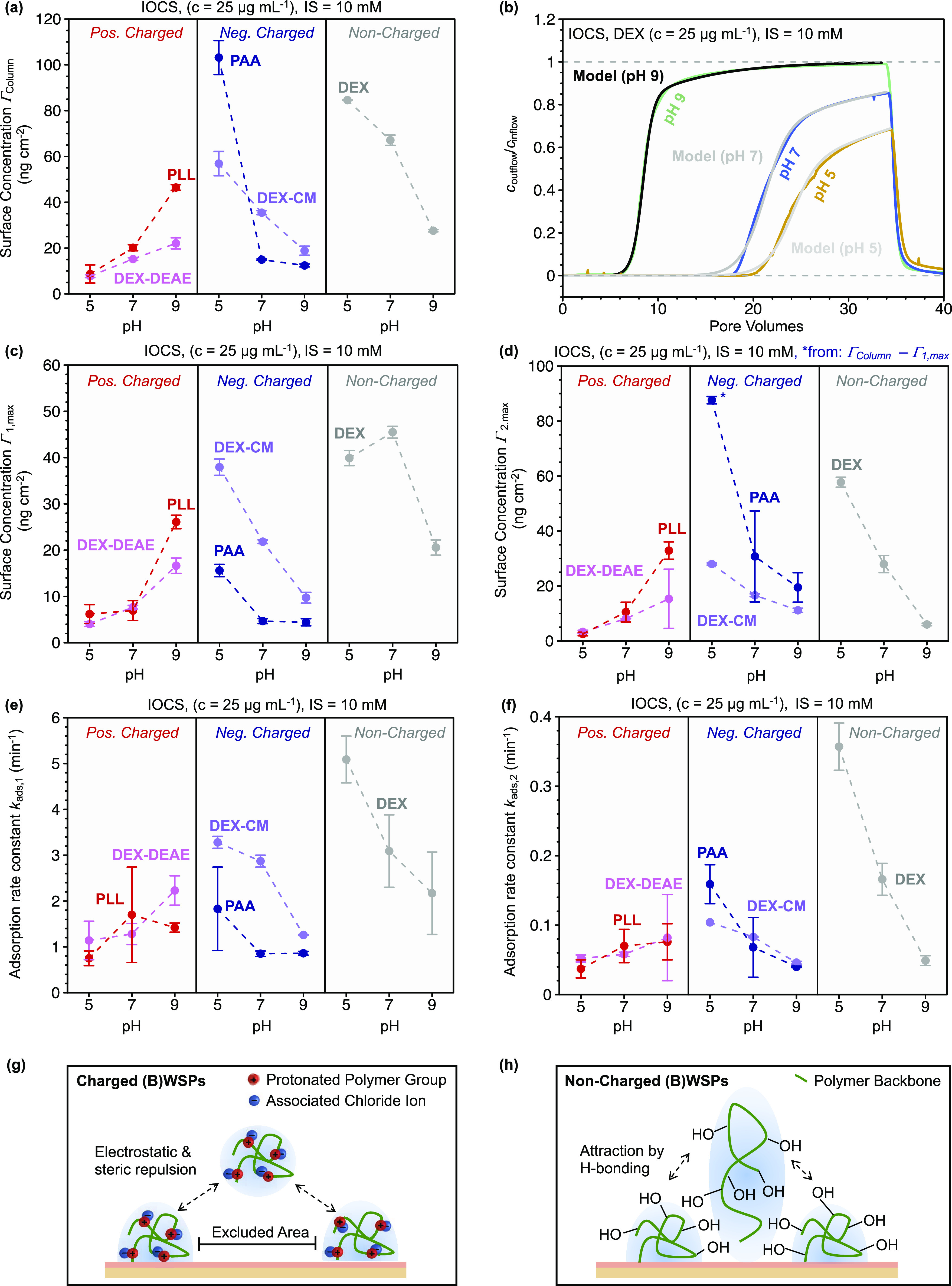
Results of breakthrough experiments of (biodegradable) water-soluble polymers ((B)WSPs) through columns filled with iron oxide-coated sand (IOCS). The (B)WSPs are diethylaminoethyl dextran (DEX-DEAE), poly-l-lysine (PLL), carboxymethyl dextran (DEX-CM), poly(acrylic acid) (PAA), dextran (DEX), and polyethylene glycol monomethyl ether (mPEG). (a) Effect of solution pH on the final adsorbed concentrations of (B)WSPs on the IOCS surface determined by mass balance calculations as a function of pH (at constant ionic strength, IS = 10 mM). (b) Effect of solution pH on DEX breakthrough curves as well as fits of the transport model that included two-kinetic components in DEX adsorption. Model fits are shown in gray. *c*_outflow_ and *c*_inflow_ correspond to the concentrations of DEX in the column outflow and inflow, respectively. (c) Modeled maximum adsorption capacities of the first kinetic adsorption regime and (d) second (slower) kinetic adsorption regime as well as adsorption rate constants for (e) the first regime and (f) the second regime, all for (B)WSP adsorption to IOCS as a function of pH (at constant ionic strength, IS = 10 mM). Data points represent the mean values of duplicate measurements and fits, and error bars are the absolute deviation from the mean results propagated with modeled standard deviations via Gaussian error propagation. Schematic depictions of causes of the slow kinetic adsorption for (g) charged (B)WSPs and (h) noncharged (B)WSPs.

We conducted pH-dependent transport experiments in IOCS-packed columns for all (B)WSPs except mPEG due to its weak adsorption to IOCS. The BTCs of the (B)WSPs tested are shown in Section S6. Adsorbed (B)WSP concentrations on the IOCS at the end of the adsorption step are compiled in Figure 4a. The pH dependencies of (B)WSP adsorption in the column experiments were in very good agreement with those determined in the batch equilibration experiments (Figure 3a). For solution pH that resulted in favorable interactions of (B)WSPs with the IOCS (i.e., PLL at pH 9, PAA and DEX-CM, and DEX at pH 5; as discussed above), adsorption was extensive and likely resulted in complete IOCS surface coverage (i.e., experimental Γ_column_ values were in good agreement with calculated Γ_HCP, max_ values). However, as compared to batch equilibration experiments with IOCS, adsorbed concentrations of charged (B)WSPs in the column experiments were lower except for adsorption of PAA at pH 5. Smaller Γ_column_ than Γ_batch_ values may have had several causations. First, a fraction of the IOCS particle surfaces in the column may not have been available for polymer adsorption, for instance, due to adsorbed polymers blocking access to part of the interparticle pore space. Second, slower adsorption kinetics in the column may have resulted in adsorbed polymers adopting very flat and extended conformations, thereby increasing their molecular footprints and decreasing overall adsorbed amounts at surface saturation. In contrast, fast adsorption of polymer molecules in batch equilibration reactors likely resulted in more compact and coiled conformations, as unfolding of individual polymer molecules into extended confirmations on the surface was impaired by neighboring polymer molecules that already occupied the space required for unfolding. Decreasing extents of “unfolding” on the surface with increasing overall adsorption rates are well established for proteins^76,77^ and were shown to result in more compact adlayers with higher adsorbed masses at surface saturation. Third, given that polymer adsorption in the column experiments had a slow kinetic component (see below), measured adsorbed amounts at the end of the column experiments underestimated the maximum equilibrium adsorbed amounts that would have been attained had the duration of the columns experiments been extended. All measured pH-dependent maximum adsorbed surface concentrations of (B)WSPs on sand and IOCS from batch equilibration, column transport, and OWLS experiments are compiled in Table S4.

The shape of the BTCs provides information about adsorption kinetics. As discussed above for transport through sand-filled columns, the shapes of the BTCs of several (B)WSPs in IOCS-filled columns had two distinct phases, as shown representatively in Figure 4b for DEX: in the first phase, which included an initial delay in the onset of the polymer breakthrough, *c*_outflow_/*c*_inflow_ increased steadily with an approximately constant slope. In the second phase, BTC flattened: *c*_outflow_/*c*_inflow_ increased less steeply and only gradually approached unity. In some systems, the increase in *c*_outflow_/*c*_inflow_ in the second phase was so slow that breakthrough was incomplete when we initiated rinsing after 34 PVs (i.e., *c*_outflow_/*c*_inflow_ < 1 at pH 7 and 9 in Figure 4b). The presence of the second phase implies a slow kinetic component in (B)WSP adsorption to the IOCS under flow conditions. Some of the BTCs of PLL and DEX-DEAE in the sand columns also exhibited pronounced second phases and thus slow kinetic adsorption (Figure 2a,b and Section S6 for results of model fits).

For each column experiment, we fitted the (B)WSP BTCs using the *D* value obtained from the BTC of nitrate in the same column. Fitting the BTCs with an advective dispersive transport model coupled to a single-kinetic Langmuir adsorption model failed to describe the BTCs (data not shown). Instead, good fits were obtained with a two-kinetic Langmuir adsorption model (eqs 5–6) (shown exemplary in Figure 4b for DEX), allowing us to estimate the adsorption rate constants *k*_ads,1_ and *k*_ads,2_ and maximum adsorbed surface concentrations Γ_1, max_ and Γ_2, max_ for the faster (subscript 1) and slower (subscript 2) adsorption kinetic component. The fitted Γ_1, max_ and Γ_2, max_ values (Figure 4c,d) showed the same pH dependence as the experimentally determined Γ_column_ values (Figure 4a). Furthermore, the fitting revealed that changes in Γ_1, max_ and Γ_2, max_ were positively related, suggesting that the importance of the slow kinetic regime scaled with the maximum adsorbed concentrations in the fast kinetic regime.

For each (B)WSP, *k*_ads,1_ and *k*_ads,2_ values showed the same pH dependence as the corresponding Γ_1, max_ and Γ_2, max_ values (comparison of Figure 4e,f versus Figure 4c,d), suggesting that adsorption rates and extents in both kinetic regimes scaled. More importantly for the discussion of adsorption kinetics, *k*_ads,1_ was more than 10-fold higher than *k*_ads,2_ (Figure 4e,f) (i.e., *k*_ads,1_ ranged from approximately 1 to 5 min^–1^ while *k*_ads,2_ ranged from approximately 0.04 to 0.4 min^–1^). These rate constants imply that adsorption of (B)WSPs in the fast kinetic regime occurred on the time scale of a few minutes, whereas adsorption in the slow kinetic regime occurred over tens of minutes to a few hours.

The second phase of slow adsorption kinetics may have been linked to coverage of the sorbent surface by (B)WSP molecules adsorbed in the first phase. This coverage likely led to electrostatic and steric repulsion between the adsorbed and dissolved (B)WSPs, thereby slowing down further adsorption and giving rise to the “excluded-area effect” (see Figure 4g for a schematic depiction). Uncharged (B)WSPs may experience strong attractive intermolecular forces due to H-bonding (DEX) and possibly also the hydrophobic effect (PLL, pH 9).^78−80^ It is conceivable that DEX molecules that were adsorbed in the initial kinetic phase (i.e., phase 1, fast adsorption) had two effects: they sterically impaired adsorption of additional DEX molecules to the sorbent surface, while at the same time, they may have enabled slow additional adsorption of DEX molecules by allowing for H-bonding with the already adsorbed DEX molecules (i.e., kinetic phase 2, slow adsorption). The continuous adsorption of DEX may result in adlayer architectures in which DEX–DEX H-bonding plays a large role besides DEX-IOCS H-bonding. Favorable DEX–DEX interactions may explain maximum adsorbed surface concentrations Γ_i, max_ exceeding the theoretical values Γ_HCP, max_ (see Figure 4h for a schematic depiction).

### Environmental Implications

Our work provides evidence for electrostatics controlling the adsorption of charged (B)WSPs to charged mineral sorbents. Electrostatics also determine the conformations and packing densities of charged (B)WSP in adsorbed states and, thereby, the maximum adsorbed (B)WSP concentrations at the sorbent surface saturation. By comparison, H-bonding drives adsorption of noncharged (B)WSPs, with more extensive adsorption for H-bond donating and accepting (B)WSPs than for only H-bond accepting (B)WSPs. Our findings support that electrostatic and H-bond interactions also control (B)WSP adsorption to mineral surfaces in soils.

For all tested (B)WSPs and both sorbents, we found very good agreement in the trends in adsorption with pH and IS across the four different experimental approaches (i.e., batch equilibration reactors, two surface adsorption techniques, and column transport experiments). The good agreement strongly suggests that future batch equilibration and column transport studies with natural soils as sorbents will provide consistent information about (B)WSP adsorption. Given that batch equilibration studies are more readily conducted, these may be used to screen soils to identify which soils are most interesting for testing in column studies. Our work highlights the importance of slow adsorption kinetics of (B)WSP to the mineral soil surface under hydrodynamic flow. Slow adsorption kinetics in soils may lead to enhanced vertical transport of (B)WSPs into the soil with infiltrating (rain)water, particularly during high precipitation events with high infiltration. At the same time, slow adsorption kinetics imply that BWSPs may remain in soil porewater for a sufficiently long time to undergo enzymatic breakdown and, ultimately, biodegradation. The information on (B)WSP adsorption herein also has implications for application domains of (B)WSPs beyond agriculture, including their use in oil and gas extraction and corrosion inhibition. For (B)WSPs released in these applications, adsorption to environmental particle surfaces is also a key fate process. The understanding of adsorption rates and extents of chemically diverse BWSPs to mineral surfaces provided herein will guide future studies in assessing the effects of BWSP adsorption on the biodegradation dynamics of these molecules in the environment.

## Supporting Information Available

Details on the chemicals, sorbents, and polymers used, experimental details, including a flow sheet of the column breakthrough setup, and details on the adsorption models (PDF)
