# Supplementary material for: Molecular Structure and Conformation of Biodegradable Water-Soluble Polymers Control Adsorption and Transport in Model Soil Mineral Systems
Source: Environ Sci Technol. 2024 Jan 2;58(2):1274–86. doi: 10.1021/acs.est.3c05770 (PMC10795197; doi:10.1021/acs.est.3c05770)
Supplement: Supplementary file 1 — es3c05770_si_001.pdf [file es3c05770_si_001.pdf]

Supporting information

Molecular structure and conformation of biodegradable water-soluble polymers control adsorption and transport in model soil mineral systems

Kevin Kleemann[†], Patrick Bolduan[‖], Glauco Battagliarin[‖], Iso Christl[†], Kristopher McNeill[†] and Michael Sander[†*]

[†]Institute of Biogeochemistry and Pollutant Dynamics, ETH Zurich, 8092 Zurich, Switzerland

[‖]BASF SE, Carl-Bosch-Strasse 38, 67056 Ludwigshafen, Germany

In preparation for submission to *Environmental Science and Technology*

*Corresponding author:

Michael Sander

Email: michael.sander@env.ethz.ch

Phone: 0041- (0) 44 632 8314

Number of pages: 16

Number of figures: 13

Number of tables: 4

Section S1. Chemicals

Information on the purities and the suppliers of chemicals used are provided in **Table S1**.

Table S1. Overview of chemicals used, their purities and suppliers.

Chemical	Purity (%)	Supplier
1-ethyl-3-(3-dimethyl-aminopropyl) carbodiimide hydrochloride (EDAC)	Not specified (Commercial grade)	Sigma Aldrich
1,10-phenanthroline hydrochloride monohydrate	≥ 99.5	Sigma Aldrich
3-(*N*-morpholino)propane sulfonic acid (MOPS)	> 99	VWR
6-amino fluorescein	94.5	Sigma Aldrich
Hydrochloric acid (37%)	> 99	Sigma Aldrich
N, N-Diethyl piperazine (DEPP)	98	Thermo Scientific
Sodium hydroxide	> 98	Sigma Aldrich
Sodium nitrate	≥ 99.5	Sigma Aldrich
Sodium chloride	> 99	Sigma Aldrich

Section S2. Polymers

The properties of polymers used are listed in **Table S2**.

Table S2. Overview of polymers used in this study and their physicochemical properties.

**Polymer Type** Fluorescein isothiocyanate (FITC) functionalized	**Molecular weight, M_w (kDa)**	**FITC Labeling Degree (mol% of funct. monomers)**	**Functionalization Degree (mol% of funct. monomers)**	**Acidity constant, pK_a**
Dextran-FITC (DEX)	36.4[a,b]	0.1 – 2[b]	/	/
Diethyl aminoethyl dextran-FITC (DEX-DEAE)	approx. 40[b]	0.5[b]	25 – 30[c]	5.5[c] and 9.2[c]
Carboxymethyl-dextran-FITC (DEX-CM)	50.1[a,b]	0.4[b]	5.7[b]	6.1[1]

Methoxy polyethylene glycol-FITC (mPEG)	10[d]	0.4[d]	/	/
Poly-L-lysine-FITC (PLL)	25.5[b]	0.6[b]	/	10.44[2]
Poly aspartic acid-FITC (PAA)	8[b]	0.7	/	4.7[3]

a Determined by gel permeation chromatography (GPC) analysis; b According to the Sigma Aldrich analysis certificate of the used batch; c Determined by acid-base titration (Figure S1); d According to the creativePEGworks analysis sheet

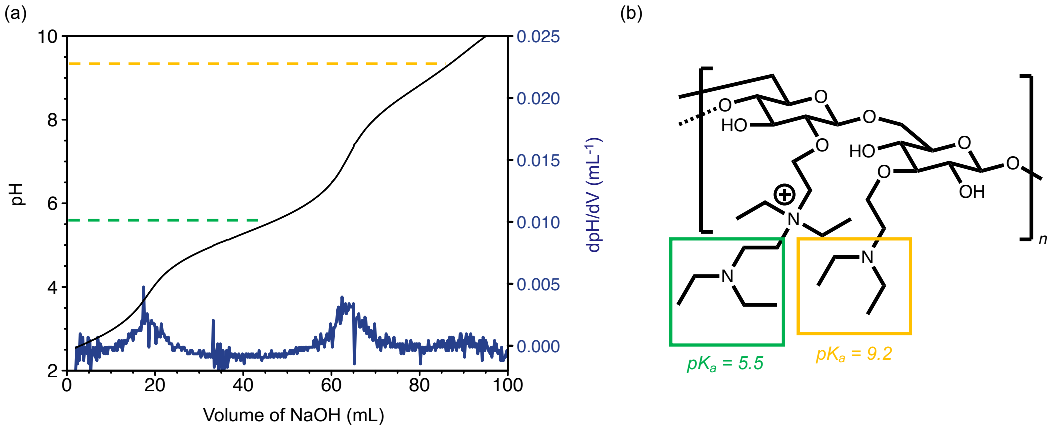

Figure S1. (a) Acid-base titration data for diethyl aminoethyldextran-FITC (DEX-DEAE) and **(b)** schematic chemical structure of two types of ionizable DEAE groups.

Section S3. Sorbents

Iron oxide-coated sand (IOCS). The sand (Sigma Aldrich, Switzerland, acid-washed and calcined, ≥ 99.7%) was coated with iron oxide using a method established by Mills et al.[4]. The first step involved cleaning the sand. In brief, we reacted 100 g of sand with 90 mL of a 10% nitric acid solution for 2 hours in an Erlenmeyer flask placed on an orbital rotary shaker (250 rpm). We subsequently decanted the supernatant and rinsed the sand with MQ water until the liquid was transparent. Next, the MilliQ water was decanted from the flask, and 90 mL of a 0.5 M NaOH solution was added to the sand, followed by shaking the sand on an orbital rotary shaker (250 rpm) for another 2 hours. We thoroughly rinsed the sand with MilliQ water and air-dried the sand overnight at 90°C.

We transferred 150 g of the cleaned sand (as described above) to a Nalgene bottle containing 300 mL of a 1 M $FeCl_3$ * 6 H_2O solution. The Nalgene bottle was then placed in a heating block and maintained at a constant temperature of 30°C. The suspension was stirred continuously using an overhead stirrer (uniSTIRRER OH_2, LLG Labware, Germany). We adjusted the pH of the suspension from pH 1.2 to pH 5.5 by using a 2.5 M NaOH solution,

leading to the precipitation of Fe(III) oxyhydroxide on the sand surface. The suspension was aged under vigorous stirring at this pH and temperature for over 55 hours. The iron oxide-coated sand (IOCS) was then washed thoroughly with MilliQ water until the supernatant was clear, and then transferred to a filter paper (Macherey-Nagel MN615) and washed again with MilliQ water until the filtrate was clear. We dried the IOCS in an oven at 60°C for at least 24 hours before mixing and homogenizing the IOCS from various coating batches to be used in column transport experiments. Using 1,10-phenanthroline hydrochloride monohydrate as a complexing agent, we colorimetrically measured the total iron concentrations on the surfaces of IOCS.[5] Absorbance was measured at a wavelength of 510 nm using a microplate absorbance reader (Infinite M Nano, Tecan, Switzerland).

The pH-dependent surface charge and the point of zero charge of the sand and IOCS were determined using a computer-controlled potentiometric acid-base titration system[6] from pH 4 to pH 10 and at two ionic strengths of IS = 0.01 M and 0.1 M NaCl, as shown in **Figure S2**.

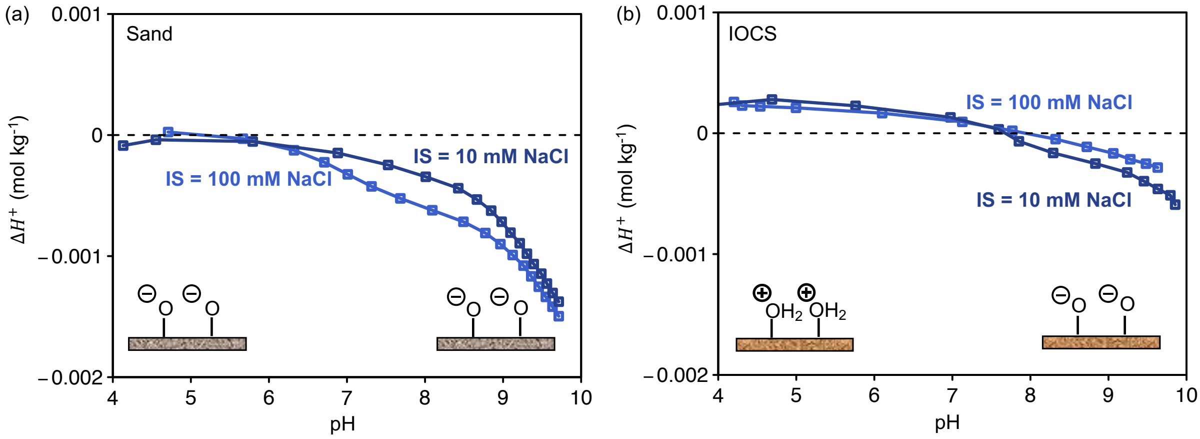

Figure S2. Acid-base titration of **(a)** sand and **(b)** the iron oxide-coated sand (IOCS) in ionic strengths of IS= 0.01 M and 0.1 M, set by NaCl as background electrolyte.

Section S4. Quartz Crystal Microbalance with Dissipitation monitoring

QCM-D determines changes in adsorbed masses on the surfaces of QCM-D sensors by monitoring the resulting mass-dependent changes in the resonance frequencies of piezoelectric quartz oscillators that are integrated into these sensors. We used a peristaltic pump to deliver polymer solutions (5 µg mL^{-1}) and buffer solutions at a constant volumetric flow rate of 20 µL min^{-1} at room temperature to the sensor cells. To obtain stable baseline readings, the sensors were equilibrated to polymer-free buffer solution for four to six hours under continuous flow.

We subsequently delivered polymer-containing solutions to the cells for 60 minutes, followed by rinsing the sensors by delivering polymer-free solutions for 30 min. The results of the QCM-D experiments for PLL are shown in **Figure S3**, while the results for DEX-DEAE are shown in **Figure 1** in the manuscript.

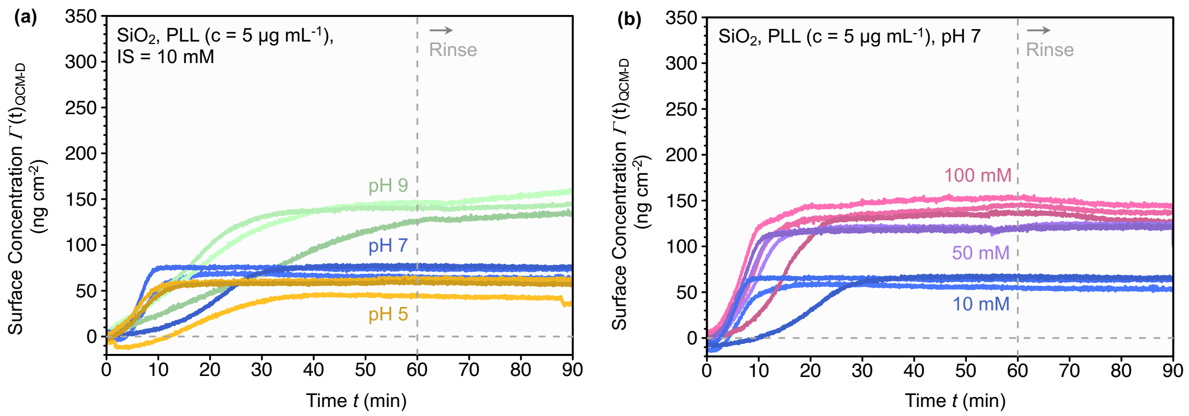

Figure S3. Changes in adsorbed surface concentration of poly-L-lysine (PLL) on SiO_2 sensors in the Quartz Crystal Microbalance with Dissipitation monitoring (QCM-D) over time at **(a)** different solution pH (ionic strength IS = 10 mM) and **(b)** ionic strengths (all at pH 7).

Section S5. Optical Waveguide Lightmode Spectroscopy (OWLS)

OWLS determines surface adsorbed concentration at a waveguide-water interface. This technique relies on grating-assisted coupling of light into an optical waveguide layer and guidance within.[7] The in-coupling angles of the laser light are sensitive to the refractive index and the thickness of adlayers on the waveguide-water interface. Changes in the in-coupling angle are monitored over time. The waveguides are mounted in flow cells.

All sensors were equilibrated with buffer solution for > 12 h prior to use. Fresh buffer solution was pumped into the flow cells for five minutes to ensure stable baseline readings, prior to delivering PLL or DEX-DEAE containing solutions. For each experiment, we delivered PLL or DEX-DEAE containing (5 µg mL^{-1}) pH-buffered solutions (i.e., *N*,*N*-diethyl piperazine (3 mM) at pH 5 and 9 and 3-(*N*-morpholino)propane sulfonic acid (3 mM) at pH 7) through a syringe at room temperature to the flow cells, followed by monitoring PLL or DEX-DEAE adsorption in batch mode (no flow through the sensor cell). Subsequently, we delivered fresh

polymer solution to the cell for at least five minutes to ensure that stable final readings were obtained also when re-delivering polymer to the cell. Afterwards, the flow cells were rinsed continuously with polymer-free buffer solution for 5 minutes to assess adsorption reversibility. The results the OWLS experiments for DEX-DEAE and PLL are shown in **Figure S4**.

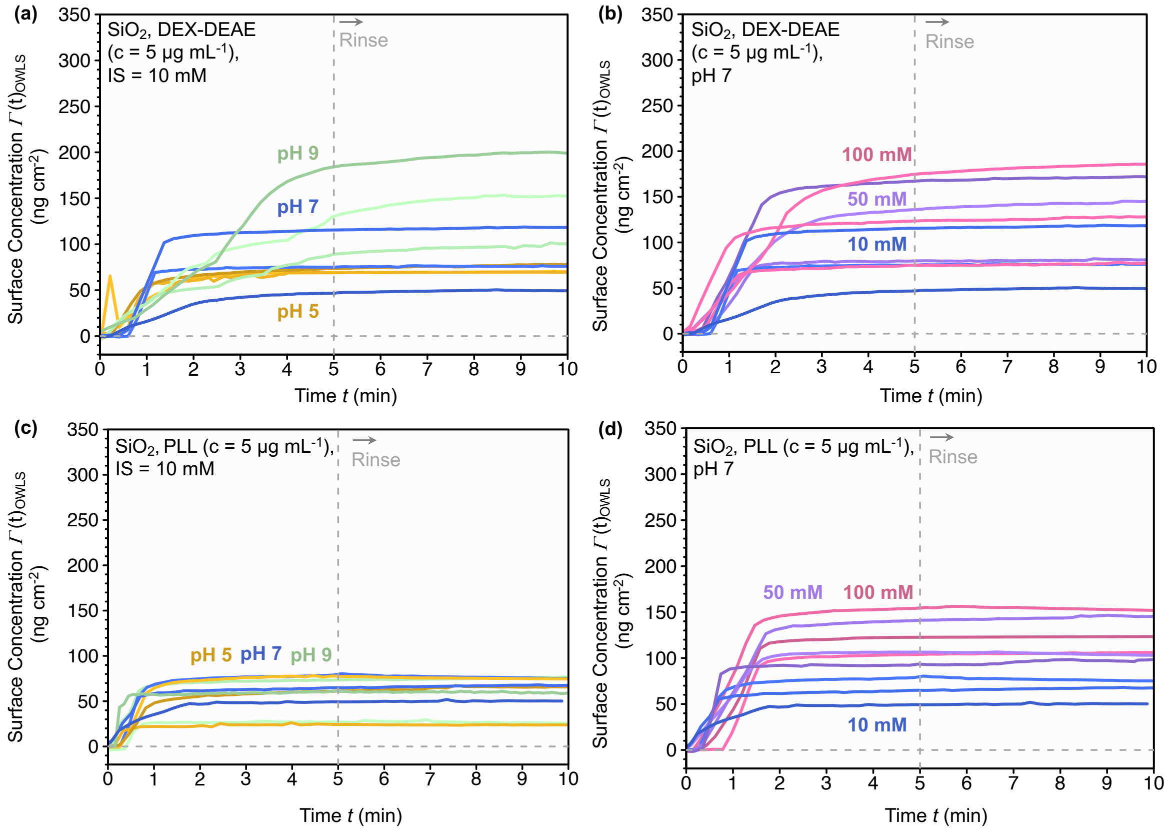

Figure S4. Changes in adsorbed surface concentrations of polymers on silica (SiO_2) sensors over time determined by Optical Waveguide Lightmode Spectroscopy (OWLS) for **(a, b)** diethyl aminoethyl dextran (DEX-DEAE) at different pH values (ionic strength IS = 10 mM; panel a) and ionic strengths (pH 7; panel b) and for **(c, d)** poly-L-lysine (PLL) at different pH values (ionic strength IS = 10 mM; panel c) and ionic strengths (IS) (pH 7; panel d).

Section S6. Column breakthrough experiments

A detailed scheme of the column setup is depicted in **Figure S5**. Borosilicate glass columns (Omnifit GmbH, Germany, inner diameter and length of 0.66 cm and 7.0 cm, respectively) were wet-packed with sorbents (i.e., sand or IOCS) in buffer solution (*N*,*N*-diethyl piperazine (3 mM for pH 5 and 9 and 3-(*N*-morpholino)propane sulfonic acid (3 mM) for pH 7) calculating a pore volume (PV) of 1.2 ± 0.05 mL (corresponding to a porosity of approx. 0.5 ± 0.02). We equipped the column end pieces with regenerated cellulose filter papers to retain potentially

mobilized colloids in the columns. Solutions were delivered to the columns from 25 mL and 50 mL syringes at a constant volumetric flow rate of 0.2 mL min^{-1} using a syringe pump system (Cetoni GmbH, Germany). To calculate the flushed PVs during one breakthrough experiment, we multiplied the flow time by the flowrate and then divided the result by the volume of one PV. The concentrations of the inert tracer nitrate in the column effluent were continuously monitored by using UV absorption measurements (λ_{ads} = 220 nm) in a spectrophotometric flow-through cell (UV Flow cell, 1/16"; Knauer, Germany) connected to a UV light source and a spectrophotometer (HL2000, Ocean Optics, USA) and by converting measured absorbance values to concentrations using nitrate calibration standards separately run through the flow cell. We determined (B)WSP concentrations in the column effluent by continuous fluorescence measurement using a fluorescence flow-through cell (SMA-FL, FIA Lab) that was fiber optically coupled to a LED light source (λ_{ex} = 470 nm ± 30 nm, 17.2 mW, 1000 mA, ThorLabs, Germany) and a spectrophotometer (Maya 2000 Pro, Ocean Optics, USA). We transformed the fluorescence signal into polymer concentrations by calibrating with standards of known concentration, separately run through the flow cell, and observing a linear relationship between fluorescence signal and concentration.

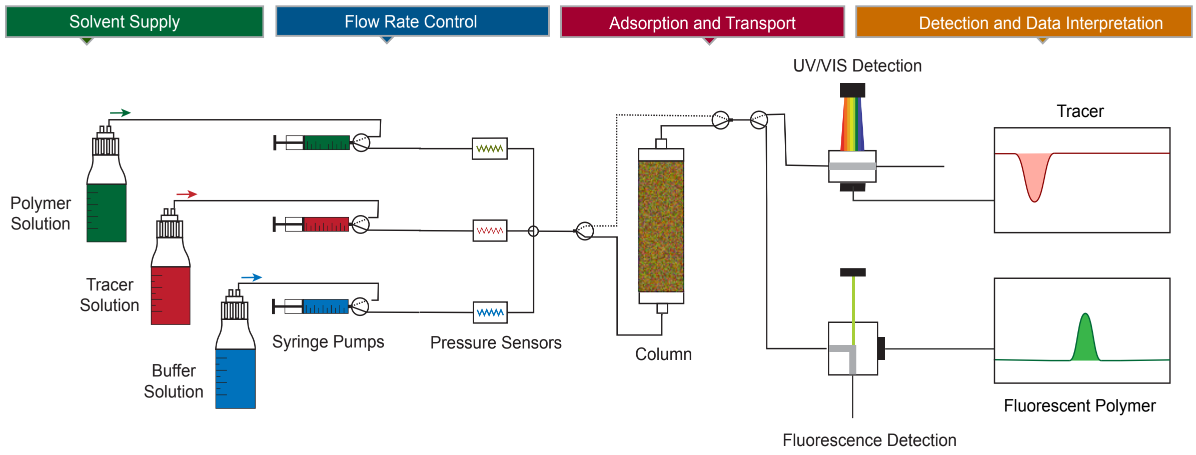

Figure S5. Schematic illustration of the column breakthrough setup.

Two representative nitrate breakthrough curves are shown in **Figure S6**. All breakthrough curves of DEX-DEAE and PLL through columns packed with sand and IOCS are shown in **Figure S7** and **Figure S8**, respectively. Adsorbed polymer concentrations on the sand and IOCS surfaces in the columns were determined by mass balance calculations according to **equation 1** and **equation 4** in the manuscript. These adsorbed concentrations are shown in **Figure S9**.

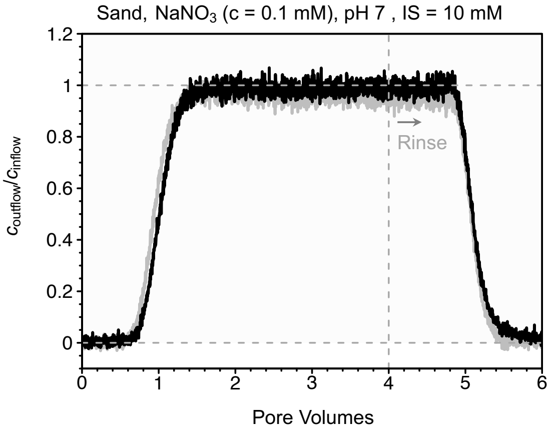

Figure S6. Two representative breakthrough curves of the conservative tracer nitrate in sand-packed columns, ran before (black line) and ran after (grey line) (B)WSP breakthrough.

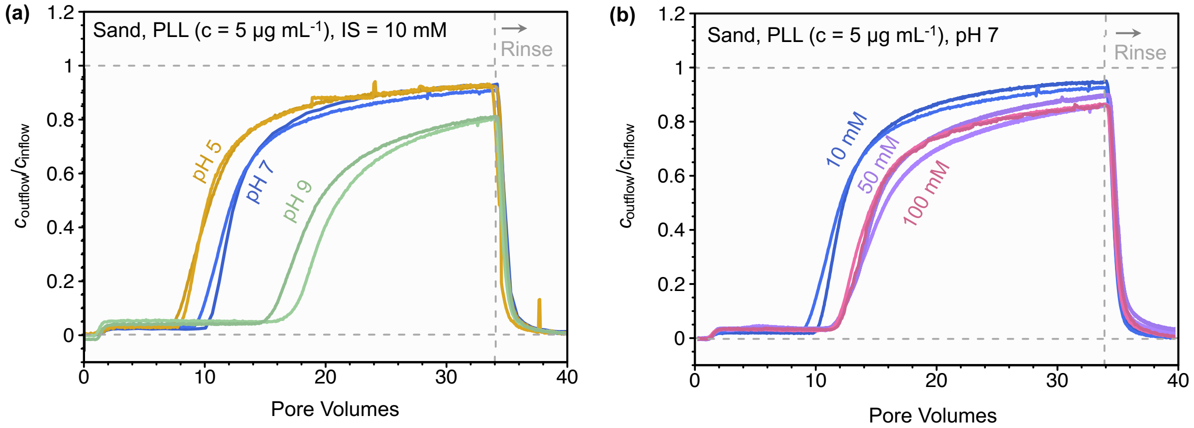

Figure S7. Breakthrough curves of poly-L-lysine (PLL) in sand-packed columns at **(a)** different pH values (ionic strength IS = 10 mM) and **(b)** ionic strengths (pH 7).

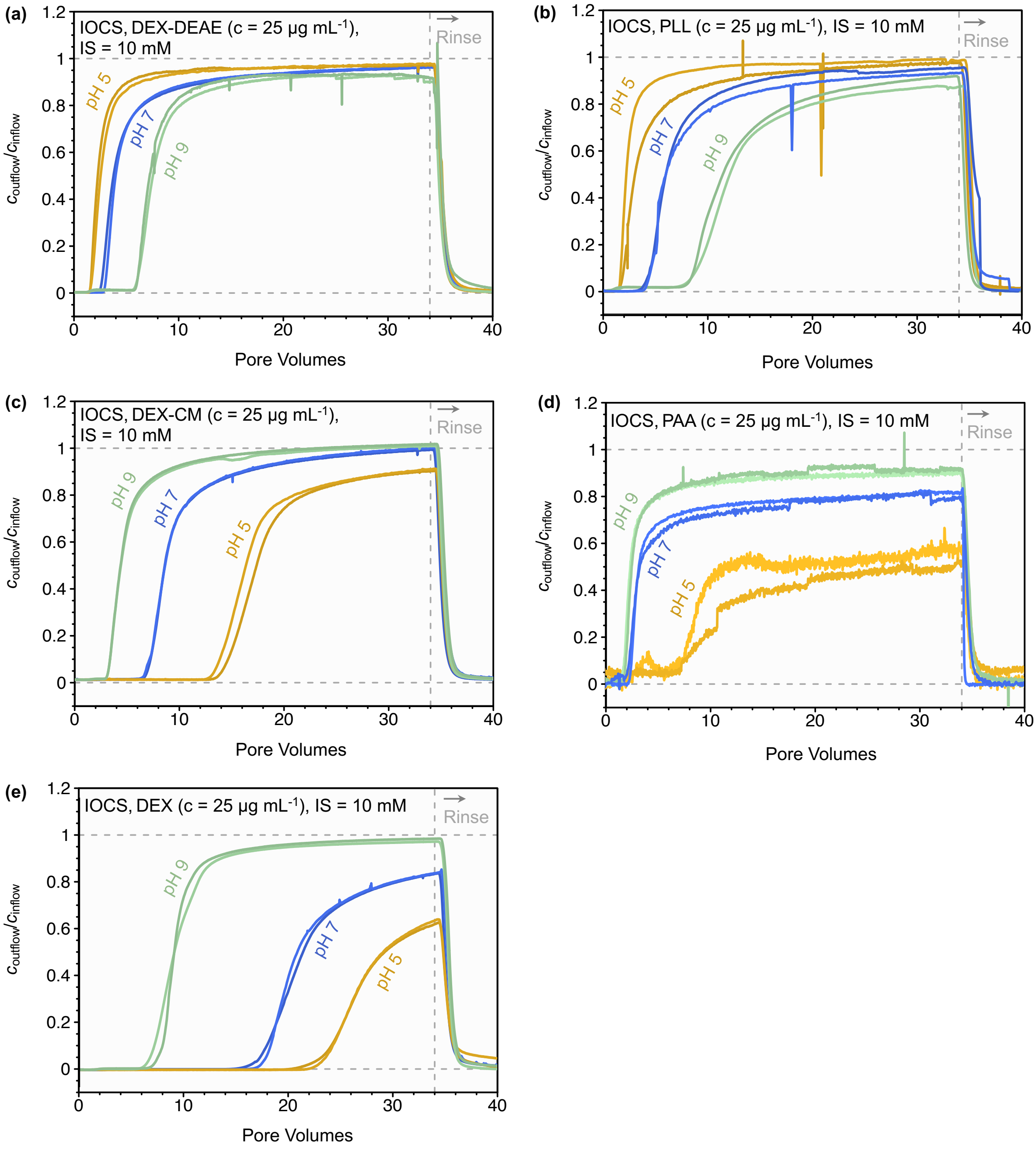

Figure S8. Breakthrough curves of **(a)** diethyl aminoethyl dextran (DEX-DEAE), **(b)** poly-L-lysine (PLL), **(c)** carboxymethyl dextran (DEX-CM), **(d)** polyacrylic acid (PAA) and **(e)** dextran (DEX) in iron oxide-coated sand IOCS columns different pH values (ionic strength IS = 10 mM). We note that occasional sharp peaks in the fluorescence readings resulted from degassing of water and formation of small bubbles in the fluorescence flow cell which, however, were then transported out of the cells.

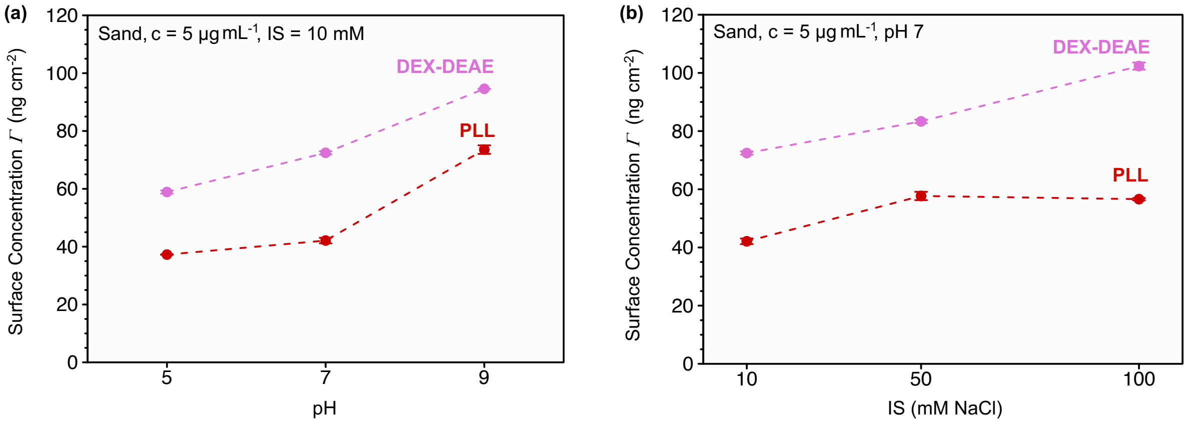

Figure S9. Dependencies of calculated maximum adsorbed concentrations of diethyl aminoethyl dextran (DEX-DEAE) and poly-L-lysine (PLL) on sand at **(a)** different solution pH values (ionic strength IS = 10 mM) and **(b)** ionic strength (IS) (pH 7), determined by mass balance calculations. Data points represent the mean values of duplicates, and error bars are the absolute deviation of these measurements from the mean.

Section S7. Adsorption and Transport Model

We modeled all breakthrough curves using Aquasim (2.1g software package)[8]. We divided the one-dimensional compartment into 190 nodes with uniform node spacing, using the 'advective-diffusive reactor compartment' option. We linked the outflow to a small-volume (0.001 mL) "mixed-reactor compartment" in order to generate flux-averaged[5] concentrations in the column outflow. Aquasim estimates the model parameters of **equation 5** and **equation 6** in the manuscript by minimizing the sum of squares of the weighted deviations $X^2(p_i)$ between calculated model and measured breakthrough curve:

$$X^2(p_i) = \sum_{l=1}^{n} \left(\frac{\left(y_{\mathrm{BTC},k} - y_k(p)\right)}{\sigma_{\mathrm{meas},k}} \right)^2 \text{ with } p_i = (k_{\mathrm{ads},1}, k_{\mathrm{ads},2}, S_{1,\mathrm{max}}, S_{2,\mathrm{max}}) \text{ and } l \in \{1, \ldots, n\} \qquad \text{Eq. S1}$$

where $y_{\mathrm{BTC,k}}$ is the value of the lth datapoint of the breakthrough curve, $\sigma_{\mathrm{meas,k}}$ is the standard deviation estimated by the program and $y_{\mathrm{k}}(p_i)$ is the calculated value of the model parameter p_{i} for the lth data point of the measurement, and n is the number of data points. We modeled the nitrate breakthrough setting $\frac{\partial S(t)}{\partial t}$ to zero in **equation 5** in the manuscript, and thereby assumed

no adsorption of nitrate. Thus, for nitrate, we minimized the sum of squares of the weighted deviations $X^2(p)$ only through variation of the hydrodynamic dispersion coefficient D. **Figures S10** and **S11** show the dependencies of modeled maximum adsorption capacities, $S_{i,max}$ and adsorption rate constants, $k_{ads,i}$, respectively, of the first (i=1) and second (i=2) kinetic adsorption regimes for polymers DEX-DEAE and PLL on sand.

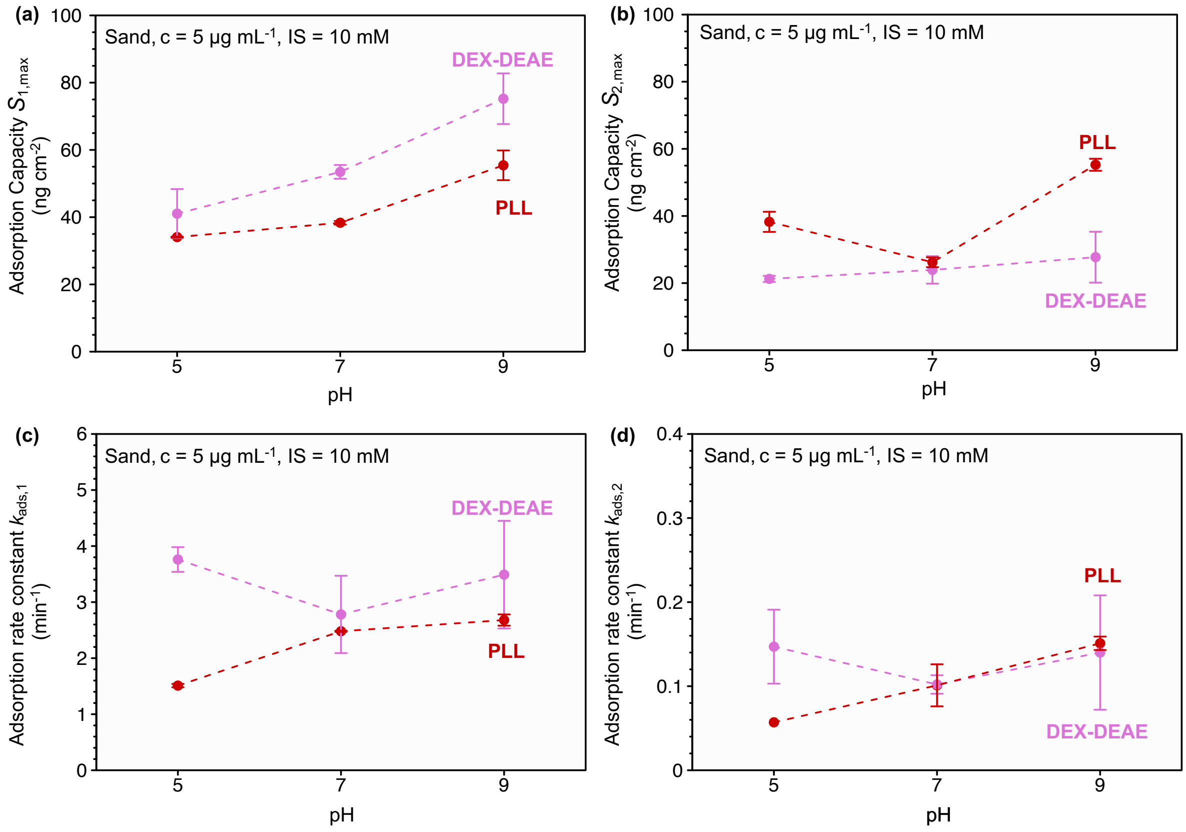

Figure S10. Dependencies of modeled maximum adsorption capacities of **(a)** the first and **(b)** the second kinetic adsorption regimes and of adsorption rate constants for the **(c)** the first and **(d)** the second kinetic adsorption regime for diethyl aminoethyl dextran (DEX-DEAE) and poly-L-lysine (PLL) adsorption to sand at different pH values (ionic strength = 10 mM). Data points represent the mean values of duplicate experiments, and error bars are the absolute deviation of single measurements from the mean propagated with modeled standard deviations via Gaussian error propagation.

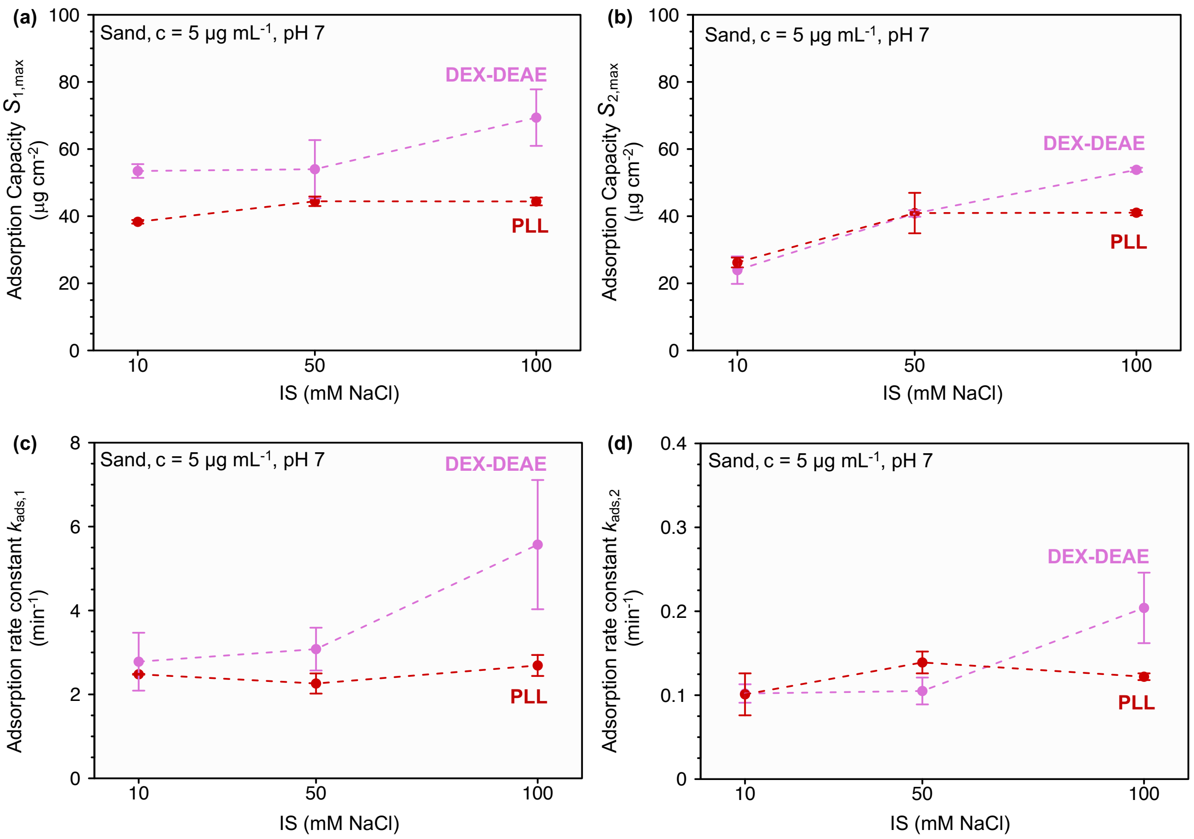

Figure S11. Dependencies of modeled maximum adsorption capacities of **(a)** the first and **(b)** the second kinetic adsorption regimes and of adsorption rate constants for the **(c)** the first and **(d)** the second kinetic adsorption regime for diethyl aminoethyl dextran (DEX-DEAE) and poly-L-lysine (PLL) adsorption to sand at ionic strengths (pH 7). Data points represent the mean values of duplicate experiments, and error bars are the absolute deviation of single measurements from the mean propagated with modeled standard deviations via Gaussian error propagation.

Section S8. Hexaconal close packing (HCP) and Random sequential adsorption (RSA) models

We used two models to estimate the maximum polymer adsorption capacities on the sorbent surface. Both models assume that adsorption stopped when a monolayer of polymers had covered the surface (i.e., assuming no adsorption of additional polymers on those adsorbed onto the sorbent). In both models, we assume that the polymers behave like hard spheres with a fixed radius R_h.

Hexagonal close packing (HCP). Assuming 2D hexagonal close packing of polymer ‘spheres’, a total area of a hexagon of $6\sqrt{3}\,R_h^2$ corresponding to the total footprint of three

polymers (blue shaded area in **Figure S12a**) results in a fractional occupied area of $\frac{\pi}{2\sqrt{3}} \approx 91\%$ (jamming limit of the HCP model, Θ_{HPC}).

Random sequential adsorption (RSA). Within the RSA model, we assume random and irreversible adsorption of the polymer 'spheres' onto the surface. the model that does not permit direct contact between polymers or allow for surface diffusion. Computer simulations of RSA predict a maximum occupied area of 54.6 % (jamming limit Θ_{RSA}, **Figure S12b**).[10]

The results of the calculations using **equation 7** for the monolayer adsorbed surface concentrations of the HCP model $\Gamma_{\mathrm{HCP,max}}$ (ng cm^{-2}) and the RSA model $\Gamma_{\mathrm{RSA,max}}$ (ng cm^{-2}), respectively, the used hydrodynamic radii of the polymers R_{h} (nm), and the molecular weights of the polymers M_w (g mol^{-1}) are summarized in **Table S3.**

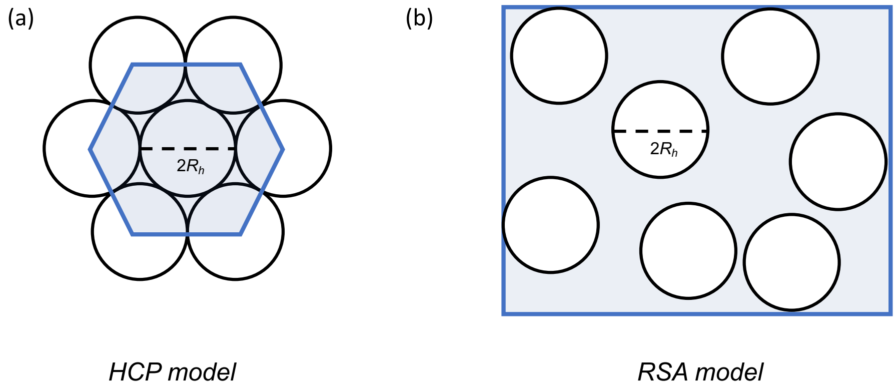

Figure S12. Schematic illustration of surface coverages for **(a)** hexagonal close packing and **(b)** random sequential adsorption of polymers as hard spheres (white circles) on a model surface area (blue areas).

Table S3. Summary of modeled monolayer adsorbed surface concentrations $\Gamma_{\text{model,max}}$, used molecular weights of the polymers M_w, and used hydrodynamic radii of the polymers R_h. The polymers are dextran (DEX), diethyl aminoethyl dextran (DEX-DEAE), carboxymethyl dextran (DEX-CM), poly-L-lysine (PLL), polyacrylic acid (PAA) and polyethylene glycol monomethyl ether (mPEG).

Polymer	$\Gamma_{\text{HCP,max}}$ (ng cm^{-2})	$\Gamma_{\text{RSA,max}}$ (ng cm^{-2})	M_w (kg mol^{-1})	R_h (nm)
DEX	76.4	46.1	36.4	4.8
DEX-DEAE	48.0	29.59	40	6.2
DEX-CM	48.0	29.5	50.1	7.1
PLL	48.0	29.5	25.5	5.1
PAA	48.0	29.5	8	2.8
mPEG	91.4	51.1	10	2.29

Table S4. Overview of the pH-dependent maximum adsorbed surface concentrations for different polymers in batch equilibration, column and optical waveguide lightmode spectroscopy (OWLS) experiments for adsorption to sand and iron oxide-coated sand (IOCS). The polymers are dextran (DEX), diethyl aminoethyl dextran (DEX-DEAE), carboxymethyl dextran (DEX-CM), polyacrylic acid (PAA), poly-L-lysine (PLL), and polyethylene glycol monomethyl ether (mPEG).

	Modeled surface concentration (column transport experiments; IOCS) (ng/cm^2)			Measured surface concentration (batch equilibration experiments; IOCS) (ng/cm^2)					
	pH 5	pH 7	pH 9	pH 5	pH 7	pH 9			
DEX	97.50 ± 1.63	73.15 ± 1.26	26.51 ± 1.64	92.94 ± 0.57	78.57 ± 0.31	53.84 ± 1.39			
DEX-DEAE	7.15 ± 0.52	15.78 ± 0.40	31.99 ± 1.65	38.46 ± 0.44	54.33 ± 1.17	73.42 ± 1.47			
DEX-CM	65.71 ± 1.75	38.41 ± 0.40	20.77 ± 1.16	78.45 ± 1.47	75.41 ± 2.28	47.88 ± 0.75			
PAA	102.88 ± 1.32	35.24 ± 0.53	23.96 ± 0.77	79.65 ± 0.59	41.82 ± 2.11	28.59 ± 0.58			
PLL	8.62 ± 2.03	17.24 ± 2.15	58.85 ± 1.45	12.95 ± 0.19	35.32 ± 7.05	80.72 ± 0.17			
mPEG				-0.87 ± 3.22	9.44 ± 1.73	9.22 ± 0.39			
	Modeled surface concentration (column transport experiments; sand) (ng/cm^2)			Measured surface concentration (batch equilibration experiments; sand) (ng/cm^2)			Measured surface concentration (OWLS experiments; silica) (ng/cm^2)		
	pH 5	pH 7	pH 9	pH 5	pH 7	pH 9	pH 5	pH 7	pH 9
DEX				6.70 ± 0.57	7.87 ± 1.41	5.55 ± 0.40			
DEX-DEAE	41.05 ± 8.20	53.47 ± 6.14	75.21 ± 14.53	45.80 ± 3.84	64.89 ± 0.65	73.17 ± 4.34	96.84 ± 45.31	98.31 ± 39.98	148.32 ± 50.122
DEX-CM				0.81 ± 1.42	5.20 ± 0.38	7.30 ± 1.43			
PAA				1.89 ± 1.41	7.47 ± 2.68	2.82 ± 1.87			
PLL	34.07 ± 3.14	38.33 ± 2.03	55.42 ± 6.21	44.87 ± 1.16	47.39 ± 1.45	64.74 ± 0.74	51.00 ± 23.12	80.00 ± 22.02	65.00 ± 19.55
mPEG				17.47 ± 3.48	20.83 ± 1.15	13.43 ± 2.68			

References

(1) Zhang, R.; Tang, M.; Bowyer, A.; Eisenthal, R.; Hubble, J. A Novel pH- and Ionic-Strength-Sensitive Carboxy Methyl Dextran Hydrogel. *Biomaterials* **2005**, *26* (22), 4677–4683. https://doi.org/10.1016/j.biomaterials.2004.11.048.

(2) Chandy, T.; Sharma, C. P. Polylysine-Immobilized Chitosan Beads as Adsorbents for Bilirubin. *Artificial Organs* **2008**, *16* (6), 568–576. https://doi.org/10.1111/j.1525-1594.1992.tb00554.x.

(3) Lee, J. W.; Kim, S. Y.; Kim, S. S.; Lee, Y. M.; Lee, K. H.; Kim, S. J. Synthesis and Characteristics of Interpenetrating Polymer Network Hydrogel Composed of Chitosan and

Poly(Acrylic Acid). *J. Appl. Polym. Sci.* **1999**, *73* (1), 113–120. https://doi.org/10.1002/(SICI)1097-4628(19990705)73:1<113::AID-APP13>3.0.CO;2-D.
(4) Mills, A. L.; Herman, J. S.; Hornberger, G. M.; Dejesús, T. H. Effect of Solution Ionic Strength and Iron Coatings on Mineral Grains on the Sorption of Bacterial Cells to Quartz Sand. *Applied and Environmental Microbiology* **1994**, *60* (9), 3300–3306. https://doi.org/10.1128/aem.60.9.3300-3306.1994
(5) Stucki, J. W. The Quantitative Assay of Minerals for Fe2+ and Fe3+ Using 1,10-Phenanthroline: II. A Photochemical Method. *Soil Sci. Soc. Am. j.* **1981**, *45* (3), 638–641. https://doi.org/10.2136/sssaj1981.03615995004500030040x.
(6) Kinniburgh, D. G.; Milne, C. J.; Venema, P. Design and Construction of a Personal-Computer-Based Automatic Titrator. *Soil Science Soc of Amer J* **1995**, *59* (2), 417–422. https://doi.org/10.2136/sssaj1995.03615995005900020021x.
(7) Tiefenthaler, K.; Lukosz, W. Sensitivity of Grating Couplers as Integrated-Optical Chemical Sensors. *J. Opt. Soc. Am. B* **1989**, *6* (2), 209. https://doi.org/10.1364/JOSAB.6.000209.
(8) Reichert, P. Aquasim - a Tool for Simulation and Data-Analysis of Aquatic Systems. *Water Science and Technology* **1994**, *30* (2), 21–30. https://doi.org/10.2166/wst.1994.0025.
(9) Parker, J. C.; van Genuchten, M. T. Flux-Averaged and Volume-Averaged Concentrations in Continuum Approaches to Solute Transport. *Water Resources Research* **1984**, *20* (7), 866–872. https://doi.org/10.1029/WR020i007p00866.
(10) Feder, J.; Giaever, I. Adsorption of Ferritin. *Journal of Colloid and Interface Science* **1980**, *78* (1), 144–154. https://doi.org/10.1016/0021-9797(80)90502-0.
